# Author Correction: Structure of the PAPP-A_BP5_ complex reveals mechanism of substrate recognition

**DOI:** 10.1038/s41467-022-33522-3

**Published:** 2022-09-28

**Authors:** Russell A. Judge, Janani Sridar, Kathryn Tunyasuvunakool, Rinku Jain, John C. K. Wang, Christna Ouch, Jun Xu, Amirhossein Mafi, Aaron H. Nile, Clint Remarcik, Corey L. Smith, Crystal Ghosh, Chen Xu, Vincent Stoll, John Jumper, Amoolya H. Singh, Dan Eaton, Qi Hao

**Affiliations:** 1 AbbVie, 1 North Waukegan Road, North Chicago, IL USA; 2 Calico Life Sciences LLC, South San Francisco, CA USA; 3 DeepMind, London, UK; 4 Department of Biochemistry & Molecular Biotechnology, University of Massachusetts Chan Medical School, Worcester, MA USA; 5 AbbVie Bioresearch Center, Worcester, MA USA; 6 Present Address: GRAIL, Menlo Park, CA USA

**Keywords:** Electron microscopy, Protein structure predictions, Proteolysis

Correction to: *Nature Communications* 10.1038/s41467-022-33175-2, Article published online 20 September 2022

In this article, the author's name Kathryn Tunyasuvunakool was incorrectly written as Kathryn Tunyasunvunakool. The original article has been corrected.

