# Supplementary material for: Structure of the PAPP-ABP5 complex reveals mechanism of substrate recognition
Source: Nat Commun. 2022 Sep 20;13:5500. doi: 10.1038/s41467-022-33175-2 (PMC9489782; doi:10.1038/s41467-022-33175-2)
Supplement: Supplementary file 3 — Description of Additional Supplementary Files [file 41467_2022_33175_MOESM3_ESM.pdf]

File name: Supplementary Data 1

Description: MD-simulation initial models

File name: Supplementary Data 2

Description: MD-simulation for final models

File name: Supplementary Movie 1

Description: Multi-body refinement analysis of PAPP-A_{BP5}. The result indicated the monomers are moving relative to one another, which explains the complex flexibility and dynamics.

File name: Supplementary Movie 2

Description: Multi-body refinement of substrate-unbound PAPP-A. The result suggested even larger movement between the two monomers compared with PAPP-A_{BP5}.
