# Supplementary material for: Structure of the PAPP-ABP5 complex reveals mechanism of substrate recognition
Source: Nat Commun. 2022 Sep 20;13:5500. doi: 10.1038/s41467-022-33175-2 (PMC9489782; doi:10.1038/s41467-022-33175-2)
Supplement: Supplementary file 8 — Reporting Summary [file 41467_2022_33175_MOESM8_ESM.pdf]

nature portfolio

Corresponding author(s): Dan Eaton, Qi Hao

Last updated by author(s): 8/24/2022

Reporting Summary

Nature Portfolio wishes to improve the reproducibility of the work that we publish. This form provides structure for consistency and transparency in reporting. For further information on Nature Portfolio policies, see our Editorial Policies and the Editorial Policy Checklist.

Statistics

For all statistical analyses, confirm that the following items are present in the figure legend, table legend, main text, or Methods section.

n/a	Confirmed	
☐	☒	The exact sample size (*n*) for each experimental group/condition, given as a discrete number and unit of measurement
☐	☒	A statement on whether measurements were taken from distinct samples or whether the same sample was measured repeatedly
☐	☒	The statistical test(s) used AND whether they are one- or two-sided<br>*Only common tests should be described solely by name; describe more complex techniques in the Methods section.*
☒	☐	A description of all covariates tested
☐	☒	A description of any assumptions or corrections, such as tests of normality and adjustment for multiple comparisons
☐	☒	A full description of the statistical parameters including central tendency (e.g. means) or other basic estimates (e.g. regression coefficient) AND variation (e.g. standard deviation) or associated estimates of uncertainty (e.g. confidence intervals)
☒	☐	For null hypothesis testing, the test statistic (e.g. *F*, *t*, *r*) with confidence intervals, effect sizes, degrees of freedom and *P* value noted<br>*Give P values as exact values whenever suitable.*
☒	☐	For Bayesian analysis, information on the choice of priors and Markov chain Monte Carlo settings
☒	☐	For hierarchical and complex designs, identification of the appropriate level for tests and full reporting of outcomes
☒	☐	Estimates of effect sizes (e.g. Cohen's *d*, Pearson's *r*), indicating how they were calculated

Our web collection on statistics for biologists contains articles on many of the points above.

Software and code

Policy information about availability of computer code

Data collection	Agilent 1200 Series Infinity II HPLC, AKTA PURE 25M, Agilent 6230 Time-of-Flight mass spectrometer, CLARIOstar 5.70R2, SerialEM V 3.9 for cryo-EM data collection
Data analysis	Astra 7.0, MassHunter B.07, cryoSPARC v3, RELION v3, Phenix v1.19.2, COOT 0.9.4.1, ChimeraX, PyMOL 1.7.7.6, Prism 9.1.2, GROMACS-2021.3, PLUMED-2.8.0 , Image Lab (2020) v6.1.0 Build 7, BOXShade Version 3.2.1, Microsoft Excel 2022, Snapgene version 5.2.1, Clustal version 1.2.4

For manuscripts utilizing custom algorithms or software that are central to the research but not yet described in published literature, software must be made available to editors and reviewers. We strongly encourage code deposition in a community repository (e.g. GitHub). See the Nature Portfolio guidelines for submitting code & software for further information.

Data

Policy information about availability of data

All manuscripts must include a data availability statement. This statement should provide the following information, where applicable:

- Accession codes, unique identifiers, or web links for publicly available datasets
- A description of any restrictions on data availability
- For clinical datasets or third party data, please ensure that the statement adheres to our policy

The data generated or analyzed in the current study are available within the article, supplementary information, or from the corresponding authors upon reasonable request. Source data are provided with this paper. The structures reported in this work have been deposited with the following accession codes: PAPP-A/IGFBP5 peptide cryo-EM structure: PDB 7UFG , EMDB EMD-26475; Substrate-unbound PAPP-A cryo-EM structure: PDB 8D8O, EMDB EMD-27253

Field-specific reporting

Please select the one below that is the best fit for your research. If you are not sure, read the appropriate sections before making your selection.

☒ Life sciences ☐ Behavioural & social sciences ☐ Ecological, evolutionary & environmental sciences

For a reference copy of the document with all sections, see nature.com/documents/nr-reporting-summary-flat.pdf

Life sciences study design

All studies must disclose on these points even when the disclosure is negative.

Sample size	Sample size calculations were not performed. Sample sizes were chosen based on pilot experiments and experimental limitations. Data variance was sufficiently low in all in vitro experiments that sample sizes did not constrain analysis. For cryo-EM samples, multiple grids were screened over several sessions to determine optimal conditions for particles with substrate-unbound PAPP-A dimer or PAPP-A/IGFBP5 complexes. For each complex, 4-8 grids were screened and 1 grid was selected for data collection for PAPP-A and 2 grids for PAPP-A/IGFBP5. Data was collected for 2-3 days for each sample based on the allotted time. The details of cryo-EM datasets including sample sizes are given in the Methods and Supplementary Information.
Data exclusions	No data were excluded from analysis. For biochemical analysis, all acquired images were processed as is usual for visual image processing. Especially for biochemical analysis, no lanes were cropped, only the edges were adjusted for better focus on the sample bands. All the raw data are provided in the source data file. For Cryo-EM, all acquired images were analyzed and micrographs with with obvious crystalline ice or empty holes were discarded for further processing.
Replication	For the biochemical analysis for activity assays (Fig. 3d,e, Fig. 4a-f, Supp. Fig. 13c, Supp. Fig. 16a-d, Supp. Fig. 17a,b, Supp. Fig. 18b,c), three independent replicates were used for analysis of all the gel- based activity assays. All replicates behaved similarly. Supplementary Fig 3e and f have two replicates to confirm reproducibility. We repeated the size-exclusion chromatography studies in Fig. 2d (n=3), and the replicates behave similarly.
Randomization	For the cryo-EM data collection, intact particles were distributed in thicker areas of ice. Particles were randomly selected from areas of thick ice for initial particle selection. For 3D refinement, all particles were randomly split into two group and processed independently. FSC measurements were determined using these independently refined half-datasets. Randomization is not applicable or relevant to biochemical assays especially protein purification, size exclusion chromatography, gel-based activity assays, fluorescent polarization, mass spectrometry.
Blinding	For Cryo-EM, all acquired images were analyzed and micrographs with with obvious crystalline ice or empty holes were excluded for further processing. Blinding was not relevant to biochemical assays especially protein purification, size exclusion chromatography, PAPP-A gel-based activity assays, fluorescent polarization, mass spectrometry. These were neither biased experiments nor based on subjective judgment but entirely based upon quantitative and qualitative measurements.

Reporting for specific materials, systems and methods

We require information from authors about some types of materials, experimental systems and methods used in many studies. Here, indicate whether each material, system or method listed is relevant to your study. If you are not sure if a list item applies to your research, read the appropriate section before selecting a response.

Materials & experimental systems

n/a	Involved in the study	
☒	☐	Antibodies
☐	☒	Eukaryotic cell lines
☒	☐	Palaeontology and archaeology
☒	☐	Animals and other organisms
☒	☐	Human research participants
☒	☐	Clinical data
☒	☐	Dual use research of concern

Methods

n/a	Involved in the study	
☒	☐	ChIP-seq
☒	☐	Flow cytometry
☒	☐	MRI-based neuroimaging

Eukaryotic cell lines

Policy information about cell lines

Cell line source(s)	Expi293FTM cells (ThermoFisher, A14528), in-house generated PAPP-A over-expression stable cell line from ThermoFisher parental HEK293-6E cell line ((ThermoFisher,11631017) Gibco 293-H cells were adapted to GibcoCD293 Medium)
Authentication	ThermoFisher STR profiling, No profiling was required for HEK293-6E as it was commercially purchased and not commonly misidentified.

Mycoplasma contamination	All cell lines tested negative for mycoplasma contamination; regular mycoplasma testing was performed
Commonly misidentified lines (See ICLAC register)	No commonly misidentified cell lines were used in this study
