# Supplementary material for: Structure of the PAPP-ABP5 complex reveals mechanism of substrate recognition
Source: Nat Commun. 2022 Sep 20;13:5500. doi: 10.1038/s41467-022-33175-2 (PMC9489782; doi:10.1038/s41467-022-33175-2)
Supplement: Supplementary file 1 — Supplementary Information [file 41467_2022_33175_MOESM1_ESM.pdf]

Supplementary information

Structure of the PAPP-A$_{BP5}$ complex reveals mechanism of substrate recognition

Russell A. Judge[1†], Janani Sridar[2†], Kathryn Tunyasunvunakool[3†], Rinku Jain[1], John C. K. Wang[2], Christna Ouch[4], Jun Xu[2], Amirhossein Mafi[2], Aaron H. Nile[2], Clint Remarcik[2], Corey L. Smith[5], Crystal Ghosh[2], Chen Xu[4], Vincent Stoll[1], John Jumper[3], Amoolya H. Singh[2,6], Dan Eaton[2*], Qi Hao[2*]

[†]Authors contributed equally to the paper
*Correspondence: qhao@calicolabs.com, deaton@calicolabs.com

Supplementary Figures 1-19 and legends
Supplementary Tables 1-2

```
Q9BXP8|PAPP-A2         NSELGWTRKKSLVEREHLNQVLLEGERCWLGAKVRRPRASPQHHLFGVYPSRAGNYLRPYPVGEQEIH
Q13219|PAPP-A     ------------------------------------------------------------------------------------------
consensus         ..........................................................................................

                                                    PAPP-A signal peptide        PAPP-A propeptide
Q9BXP8|PAPP-A2    HTGRSKPDTEGNAVSLVPPDLTENPAGLRGAVEEPAAPWVGDSPIGQSELLGDDDAYLGNQRS---KE--SLGEAGIQ----KGSAMAAT
Q13219|PAPP-A     ---------------------------------MRLWSWVLH--LGL------LSAALGCGLAERPRRARRDPRAGRPPRPAAGPATCA-
consensus         .................................     **   ...* ......  *  **       .... ..      **   .... *  *   *.

Q9BXP8|PAPP-A2 172 TTTAIFTTLNEPKPETQRRGWAKSRQRRQVWKRRAEDGQGDSGISSHFQPWPKHSLKHRVKKSPPEESNQNGGEGSYREAETFNSQVGLP
Q13219|PAPP-A   49 TRAARGRRASPPPPPPPGGAWEAVRVPR-------------------------------------RRQQREARGA-----TEEPSPPSR
consensus          *  *        * *      .*    *    *....................................  .*  .  *  .....*

                                        PAPP-A LG (2-243)
Q9BXP8|PAPP-A2  28 ILYFSGRRERLLLRPEVLAEIPREAFTVEAWVKPEGGQNNPAIIAGVFDNCSHTVSDKGWALGIRSGKDKGKRDARFFFSLCTDRVKKAT
Q13219|PAPP-A   16 ALYFSGRGEQLRLRAD--LELPRDAFTLQVWLRAEGGQRSPAVITGLYDKCSYISRDRGWVVGIHTISDQDNKDPRYFFSLKTDRARQVT
consensus           ****** * * ** ... *.**.***.  *.. ****  **.* *..* **     *.** .**..  *    .* *.**** *** .  *

Q9BXP8|PAPP-A2 118 ILISHSRYQPGTWTHVAATYDGRHMALYVDGTQVASSLDQSGPLNSPFMASCRSLLLGGDSSEDGHYFRGHLGTLVFWSTALPQSHFQHS
Q13219|PAPP-A  104 TINAHRSYLPGQWVYLAATYDGQFMKLYVNGAQVATSGEQVGGIFSPLTQKCKVLMLGGSAL--NHNYRGYIEHFSLWKVARTQREILSD
consensus          .  *  * ** *  .******  * *** * ***.* .* * . **     *. *.***    .. * .** .       *   *   *

                                                                              PAPP-A  MP(244-592)
Q9BXP8|PAPP-A2 208 SQHSSGEEEATDLVLTASFEPVNTEWVPFRDEKYPRLEVLQGFEPEPEILSPLQPPLCGQTVCDNVELISQYNGYWPLRGEKVIRYQVVN
Q13219|PAPP-A  192 METHGAHTALPQLLLQENWDNVKHAWSPMKDGSSPKVEFSNAHG--FLLDTSLEPPLCGQTLCDNTEVIASYNQLSSFRQPKVVRYRVVN
consensus               .        *.*    .. *    * * .*    *..*   ..   ..   . . * *******.*** *.*  **     *  **.** ***

                                                                                    LNR1-2 (335-394)
Q9BXP8|PAPP-A2 298 ICDDEGLNPIVSEEQIRLQHEALNEAFSRYNISWQLSVHQVHNSTLRHRVVLVNCEPSKIGNDHCDPECEHPLTGYDGGDCRLQ-GRCYS
Q13219|PAPP-A  280 LYEDDHKNPTVTREQVDFQHHQLAEAFKQYNISWELDVLEVSNSSLRRRLILANCDISKIGDENCDPECNHTLTGHDGGDCRHLRHPAFV
consensus          . .*.   ** *. **.   **   * ***    ***** * *   * **.**.*..* **. ****  . ***** * *** ******    .    .

       LNR1-2 (335-394)
Q9BXP8|PAPP-A2 387 WNRRDGLCHVECNNMLNDFDDGDCCDPQVADVRKTCFDPDSPKRAYMSVKELKEALQLNSTHFLNIYFASSVREDLAGAATWPWDKDAVT
Q13219|PAPP-A  390 KKQHNGVCDMDCNYERFNFDGGECCDPEITNVTQTCFDPDSPHRAYLDVNELKNILKLDGSTHLNIFFAKSSEEELAGVATWPWDKEALM
consensus              . *.* ..**          ** *.****  .   *   ********.***. * ***   * *   .   ***.** *   *.*** *******.*.

                                   E483
Q9BXP8|PAPP-A2 477 HLGGIVLSPAYYGMPGHTDTMIHEVGHVLGLYHVFKGVSERESCNDPCKETVPSMETGDLCADTAPTPKSELCREPEPTSDTCGFTRFPG
Q13219|PAPP-A  460 HLGGIVLNPSFYGMPGHTHTMIHEIGHSLGLYHVFRGISEIQSCSDPCMETEPSFETGDLCNDTNPAPKHKSCGDPGPGNDTCGFHSFFN
consensus          ******* * .*******  *****.** *******.*.**   ** *** ** ** ****** ** * **     * .* *   *****  *

                                                                             PAPPA  M1 + M2 (593-1133)
Q9BXP8|PAPP-A2 567 APFTNYMSYTDDNCTDNFTPNQVARMHCYLDLVYQQWTESRKPTPIPIPPMVIGQTNKSLTIHWLPPISGVVYDRASGSLCGACTEDGTF
Q13219|PAPP-A  550 TPYNNFMSYADDDCTDSFTPNQVARMHCYLDLVYQGWQPSRKPAPVALAPQVLGHTTDSVTLEWFPPIDGHFFERELGSACHLCLEGRIL
consensus           *. *.*** ** *** ***************** *  **** *. . * *.* *  *.*. * *** *  ..*  ** *  * *

Q9BXP8|PAPP-A2 657 RQYVHTASSRRVCDSSGYWTPEEAVGPPDVDQPCEPSLQAWSPEVHLYHMNMTVPCPT-EGCSLELLFQHPVQADTLTLWVTSFFM--ES
Q13219|PAPP-A  640 VQYASNASSPMPCSPSGHWSPREAEGHPDVEQPCKSSVRTWSPNSAVNPHTVPPACPEPQGCYLELEFLYPLVPESLTIWVTFVSTDWDS
consensus           **   ***   *  ** *.* ** * ***.***  *.  ***     .     .    ** . ** *** *  *.   ..**.***      ...*

Q9BXP8|PAPP-A2 744 SQVLFDTEILLENKESVHLGPLDTFCDIPLTIKLHV-DGKVSGVKVYTFDERIEIDAALLTSQPHSPLCSGCRPVRYQVLRDPPFASGLP
Q13219|PAPP-A  730 SGAVNDIKLLAVSGKNISLGPQNVFCDVPLTIRLWDVGEEVYGIQIYTLDEHLEIDAAMLTSTADTPLCLQCKPLKYKVVRDPPLQMDVA
consensus          *  . *  .*       . ***   ***.****.*  .   * *. .** **..*****.***   .***  *.*..* *.****     .

Q9BXP8|PAPP-A2 833 VVVTHSHRKFTDVEVTPGQMYQYQVLAEAGGELGEASPPLNHIHGAPYCGDGKVSERLGEECDDGDLVSGDGCSKVCELEEGFNCVGEPS
Q13219|PAPP-A  820 S-ILHLNRKFVDMDLNLGSVYQYWVITISGTEESEPSPAVTYIHGSGYCGDGIIQKDQGEQCDDMNKINGDGCSLFCRQEVSFNCIDEPS
consensus           .. *  *** *...  * .*** *.    * *  * ** .  ***  *****  .    ** ***    . *****  *  *  ***. ***

Q9BXP8|PAPP-A2 923 LCYMYEGDGICEPFERKTSIVDCGIYTPKGYLDQWATRAYSSHEDKKKCPVSLVTGEP-HSLICTSYHPDLPNHRPLTGWFPCVASENET
Q13219|PAPP-A  909 RCYFHDGDGVCEEFEQKTSIKDCGVYTPQGFLDQWASNASVSHQDQ-QCPGWVIIGQPAASQVCRTKVIDLSEGISQHAWYPCTISYPYS
consensus           **  .***.** ** **** ***.*** *.*****. *  ** * . **  .. * *. * .* .   **        .*.**  *   .
```

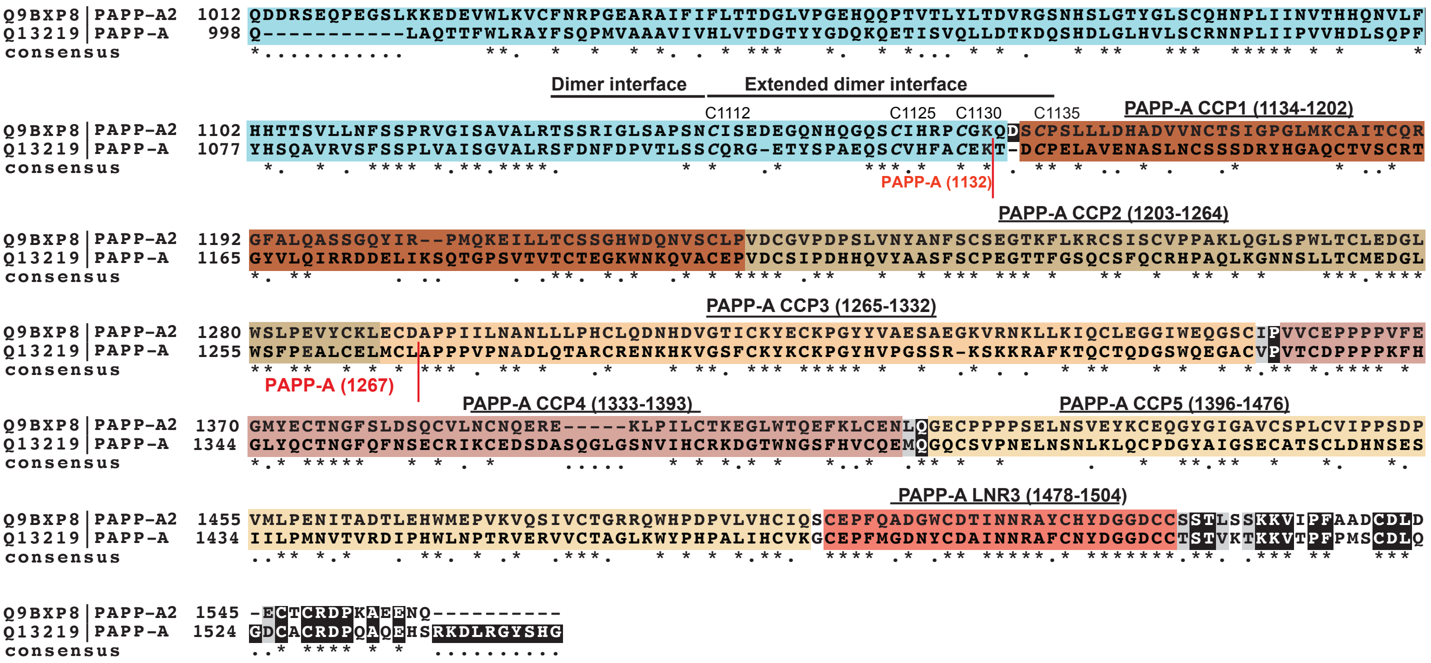

Supplementary Fig. 1: Sequence alignment of PAPP-A and PAPP-A2

Sequence alignment of PAPP-A and PAPP-A2 using Clustal Omega (version 1.2.4; Supplementary reference 3). Where the fraction of sequence conservation exceeds 0.5, residues are colored (BOXSHADE version 3.2.1). Below the sequence alignment, an asterisk “*” indicates positions that had a single, fully conserved residue and a period “.” indicates conservation between groups of weakly similar properties. Domains were delineated by color (signal peptide yellow, propeptide orange, Laminin G-like domain (LG) purple, metalloprotease domain (MP) green, central domain (M1/M2) light blue, the Complement Control Protein domains (CCP1) chocolate, (CCP2) wheat, (CCP3) peach puff, (CCP4) misty rose, (CCP5) khaki and the Lin12-Notch Repeat (LNR 3) salmon) and their name is indicated above their respective region. The active site of PAPP-A (residue E483) was highlighted in dark orange. The boundaries of two PAPP-A truncations (PAPP-A (1132) and PAPP-A (1267)) were labeled by vertical bars. Both PAPP-A and PAPP-A2 are numbered after signal sequences.

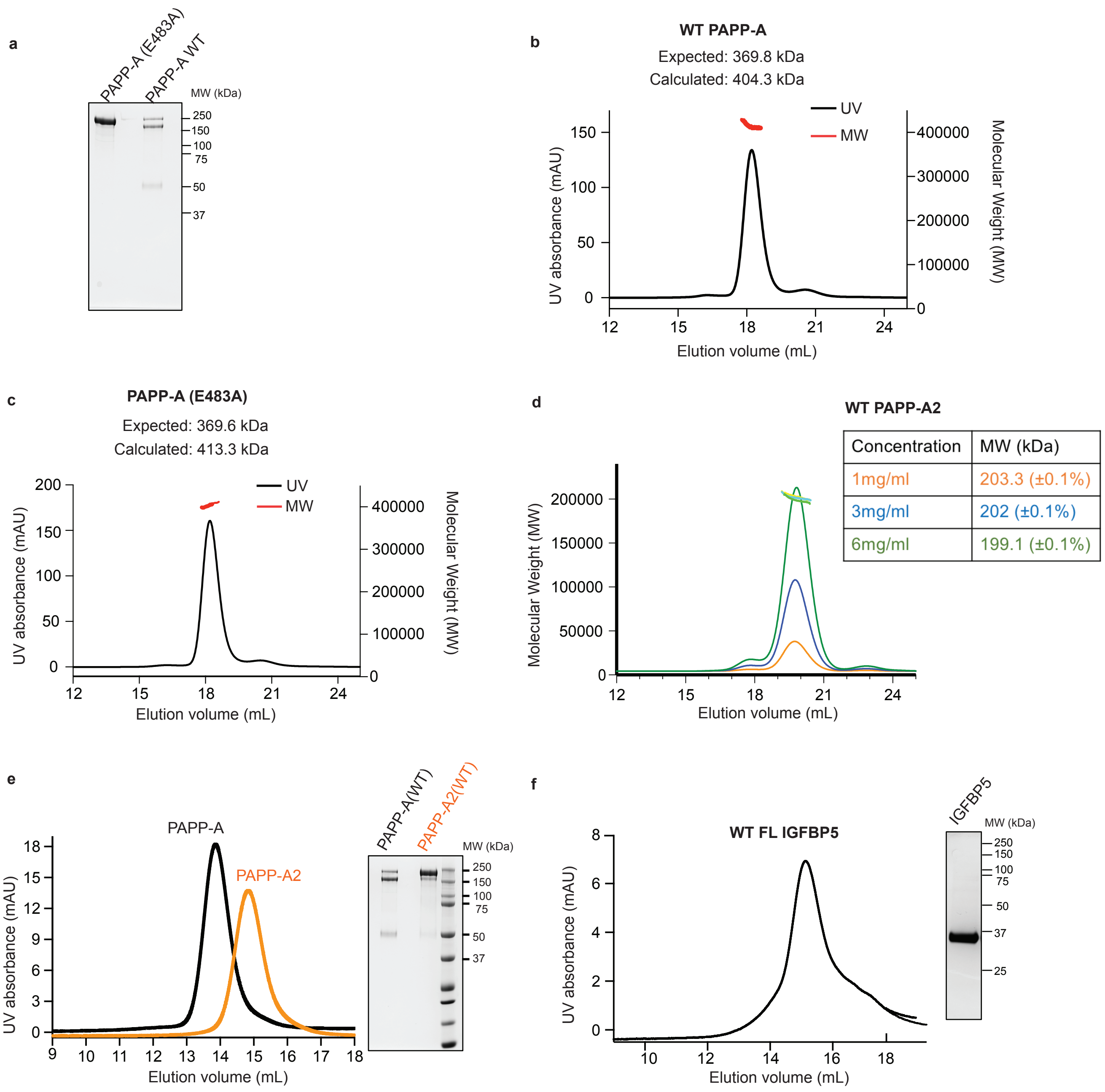

Supplementary Fig. 2: Purification and characterization of PAPP-A, PAPP-A2 and IGFBP5
a, Coomassie blue-stained SDS-PAGE gel of recombinant PAPP-A purification. The catalytic dead mutant PAPP-A (E483A) was purified as an intact protein (left lane). WT PAPP-A was purified as an auto-cleaved product (right lane), which agrees with previous reports (14, 36, 37). Autocleavage is likely enhanced due to high WT PAPP-A concentration during expression and purification. **b-c**, Multi-angle light scattering (MALS) analysis of WT PAPP-A in (**b**) and PAPP-A (E483A) in (**c**), with 1 mg/mL protein concentration. Compared with the expected homodimer size, the measured molecular weight of both proteins was ~10% higher, suggesting post-translational modifications including glycosylation. **d**, MALS analysis of WT PAPP-A2 at 3 different concentrations: 1mg/mL (orange), 3mg/mL (blue) and 6mg/mL (green). WT PAPP-A2 behaves as a monomer in solution, even at high concentration. **e**, Size-exclusion chromatography of purified recombinant WT PAPP-A (black) and WT PAPP-A2 (orange) under the same protein concentration (0.5mg/mL). UV absorbance chromatograms as well as Coomassie-stained reduced SDS-PAGE gel fractions are shown. Purified WT PAPP-A2 has limited autocleavage compared with WT PAPP-A. **f**, Size-exclusion chromatography of purified recombinant IGFBP5 protein with Coomassie-stained reduced SDS-PAGE gel of the peak fraction shown. Source data are provided as a source data file.

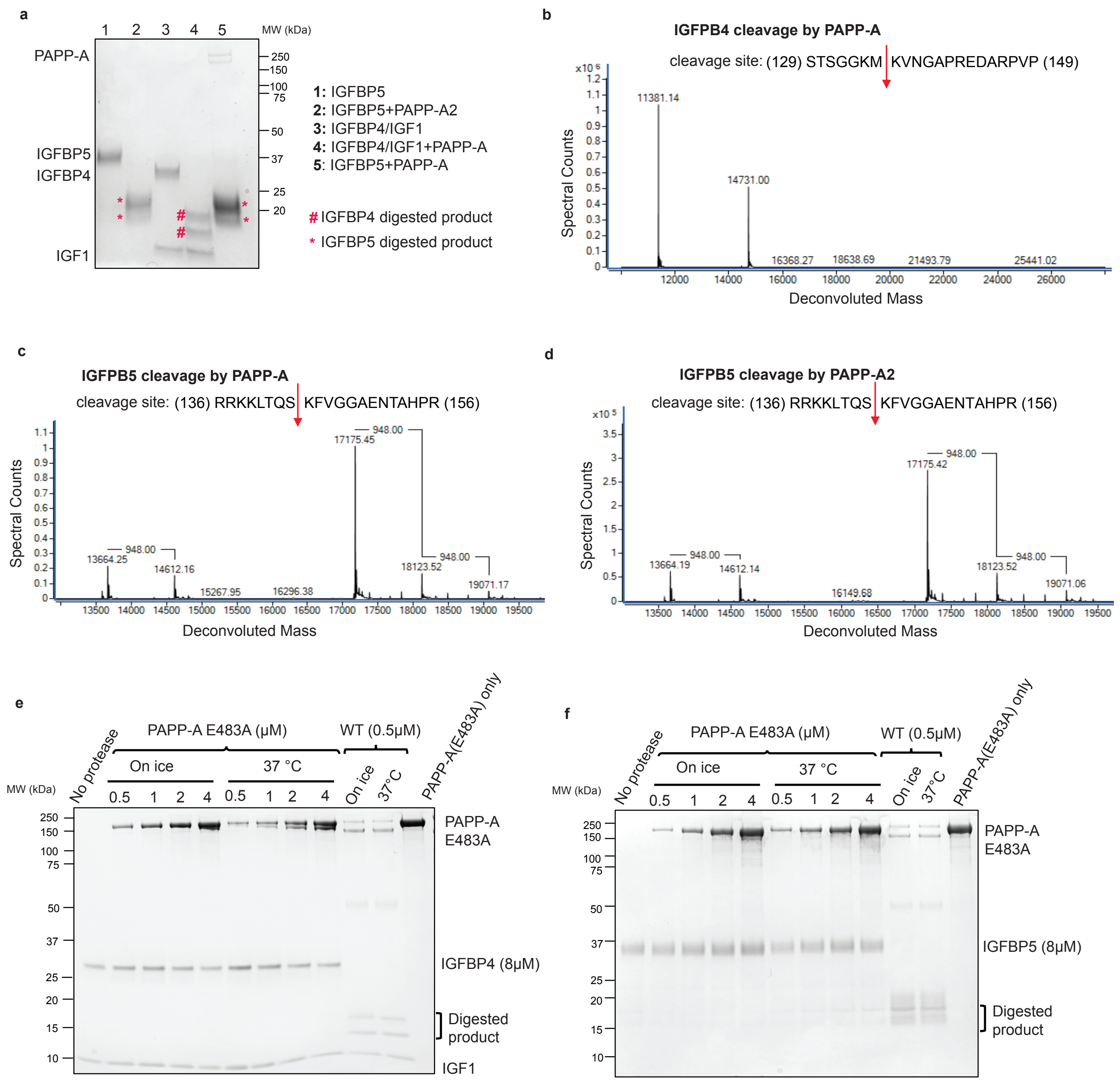

Supplementary Fig. 3: Characterization of recombinant WT PAPP-A and WT PAPP-A2 proteolytic activity towards substrates IGFBP4 and IGFBP5

a, Recombinant purified WT PAPP-A and WT PAPP-A2 proteolytic activity test with substrates IGFBP4 and IGFBP5, in the SDS-PAGE gel-based assay. IGF1 was included in the reactions with IGFBP4 to enhance cleavage efficiency. The result showed that both recombinant purified WT PAPP-A and WT PAPP-A2 cleaved IGFBP5. WT PAPP-A also fully cleaved IGFBP4 in the presence of IGF1. 500nM WT IGFBP5 or 500nM IGFBP4/500nM IGF1 were incubated with 1nM WT PAPP-A or WT PAPP-A2 and the reactions were performed at 37°C for 4 hours. **b-c**, Liquid chromatography–mass spectrometry (LC-MS) analysis of IGFBP4 (**b**) and IGFBP5 (**c**) proteolytic products by WT PAPP-A from the reaction in (**a**). **d**, LC-MS analysis of IGFBP5 proteolytic products by WT PAPP-A2 from the reaction in (**a**). WT PAPP-A specifically cleaved IGFBP4 between residues Met135 and Lys136, and both WT PAPP-A and WT PAPP-A2 specifically recognized IGFBP5 between residues Ser143 and Lys144. The red arrow in **b-d** indicated the cleavage site. This data agrees with the previous reports, and further confirmed the activity of recombinant purified WT PAPP-A and WT PAPP-A2. **e-f**, SDS-PAGE gel-based proteolytic activity assay confirmed that the catalytic dead mutant PAPP-A (E483A) is inactive. 8μM IGFBP4/IGF1 (in **e**) or IGFBP5 (in **f**) were incubated with a 2-fold serial dilution of PAPP-A (E483A) (0.5-4μM), either on ice or at 37°C for 4 hours. 0.5 μM WT PAPP-A was included in the assay as the control. Compared with the active WT PAPP-A, PAPP-A (E483A) did not show proteolytic activity for IGFBP4 and IGFBP5. The SDS-PAGE gel-based data shown in this figure are representatives of 2 independent replicates. Source data are provided as a source data file.

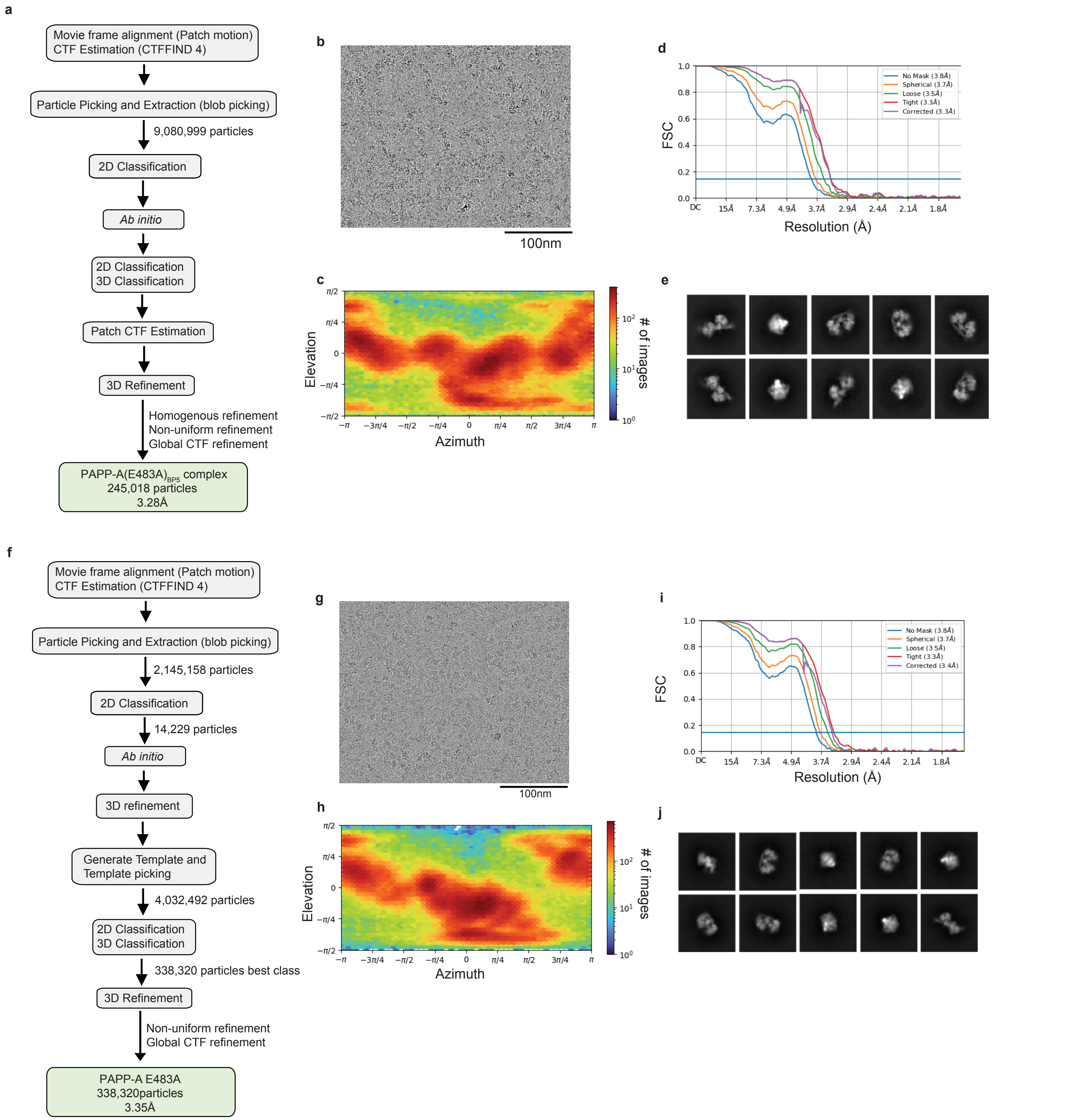

Supplementary Fig. 4: Structure and resolution determination of PAPP-A$_{BP5}$ and substrate-unbound PAPP-A (E483A)
a-e: Structure determination of PAPP-A$_{BP5}$. **a**, Flowchart summary of cryo-EM processing steps from frame alignment to 3D reconstruction. As images were taken in regions of thicker ice, coupled with extensive denaturation at the air-water interface, only ~1.3 million particles were remained after 2D classification. Further 3D classification separated out particles that were noticeably lower resolution and an additional round of 2D classification resulted in a particles stack of 245,018 images. C1 symmetry was imposed during homogeneous/heterogeneous refinement after 2 rounds of 3D classification and at all subsequent refinement steps. Note CTF is the abbreviation for Contrast Transfer Function. **b**, Example of raw images collected for PAPP-A-IGFBP5 complex sample. Scale bar is indicated. **c**, Per-particle distribution over azimuth and elevation angles (cryoSPARC v3, Supplementary reference 4). **d**, Gold-standard fourier shell correlations (FSC) for the final density map with a resolution of 3.28 Å. **e**, Representative 2D class averages for PAPP-A$_{BP5}$. **f-j**: Structure determination of substrate-unbound PAPP-A (E483A). **f**, Flowchart summary of cryo-EM processing steps from frame alignment to 3D reconstruction. C1 symmetry was imposed during homogeneous/heterogeneous refinement after 2 rounds of 3D classification and at all subsequent refinement steps. **g**, Example of raw images collected for PAPP-A (E483A) sample. Scale bar is indicated. **h**, Per-particle distribution over azimuth and elevation angles using cryoSPARC v3. **i,** Gold-standard fourier shell correlations (FSC) for the final density map with a resolution of 3.35 Å. **j,** Representative 2D class averages of substrate-unbound PAPP-A (E483A).

a

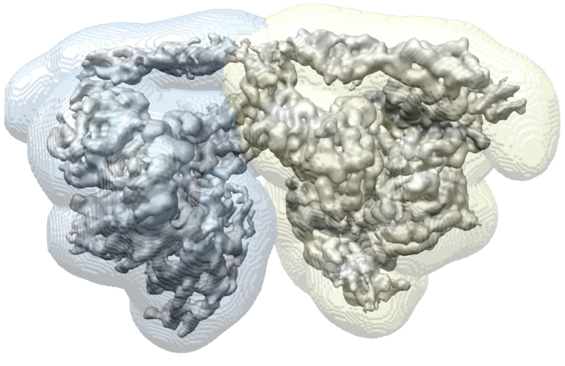

b

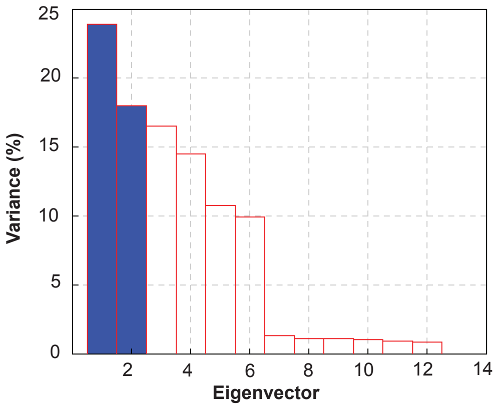

Supplementary Fig. 5: Multi-body refinement showed the two PAPP-A chains in the PAPP-A$_{BP5}$ complex twist relative to each other

a, Two soft-body masks covering each half of the density map were used for multi-body analysis. **b**, Eigenvector analysis for multi-body motion indicates that ~38% of variance is due to first 2 eigenvectors with the first 6 accounting for 80% of variance.

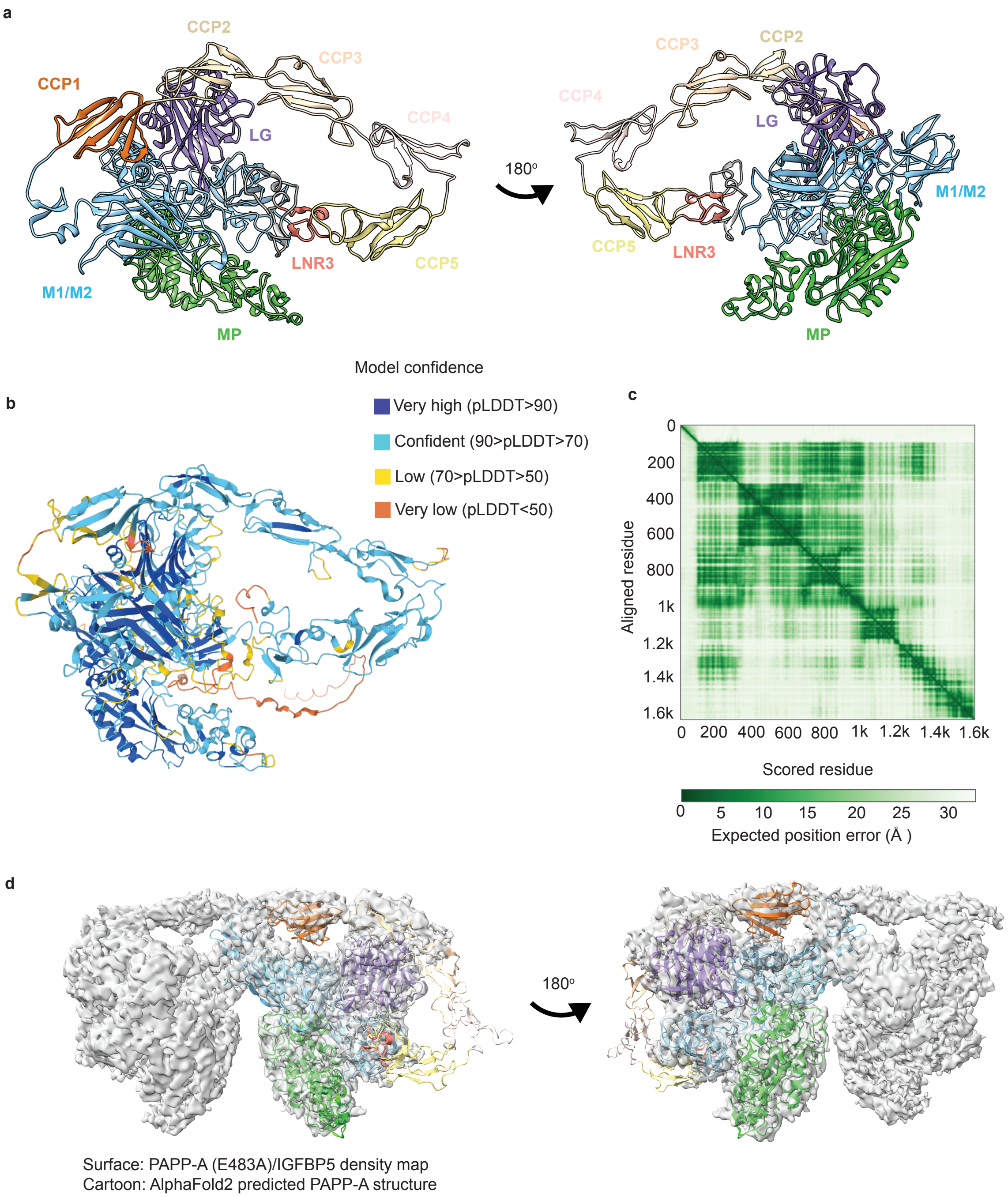

Supplementary Fig. 6: AlphaFold predicted structure facilitated the structure determination
a, Overall structure of AlphaFold predicted PAPP-A monomer. Domains are highlighted by corresponding colors as in **Fig. 1c-d**. The Laminin G-like (LG) domain, metalloprotease (MP) domain, and the central region (M1/M2) form a compact core structure. An extended C-terminal stretch of PAPP-A loops back to form an interaction between the LG and CCP2 domains. **b,** Model confidence of the AlphaFold predicted PAPP-A structure. **c,** Predicted aligned error of PAPP-A structure prediction. **b-c** are taken from AF-Q13219 (https://alphafold.ebi.ac.uk/entry/Q13219). **d,** AlphaFold predicted PAPP-A monomer structure model aligns with PAPP-A$_{\mathrm{BP5}}$ cryo-EM density map.

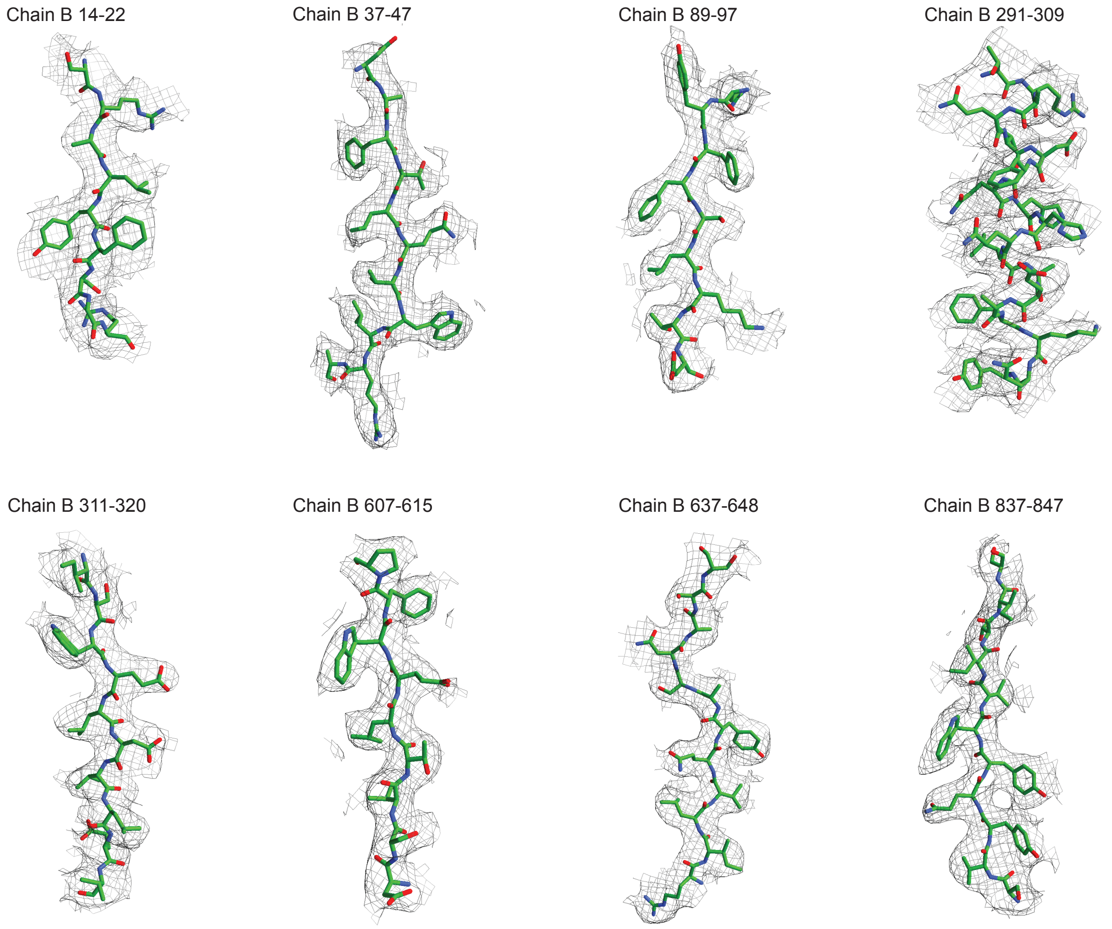

Supplementary Fig. 7: Cryo-EM density maps for selected regions of PAPP-A$_{BP5}$

Cryo-EM density (contour 0.2305) of PAPP-A chain B is depicted by the gray surface and the atomic model as green sticks. Cryo-EM density for PAPP-A$_{BP5}$ chain B core domains is well-resolved, allowing placement of residues side chains.

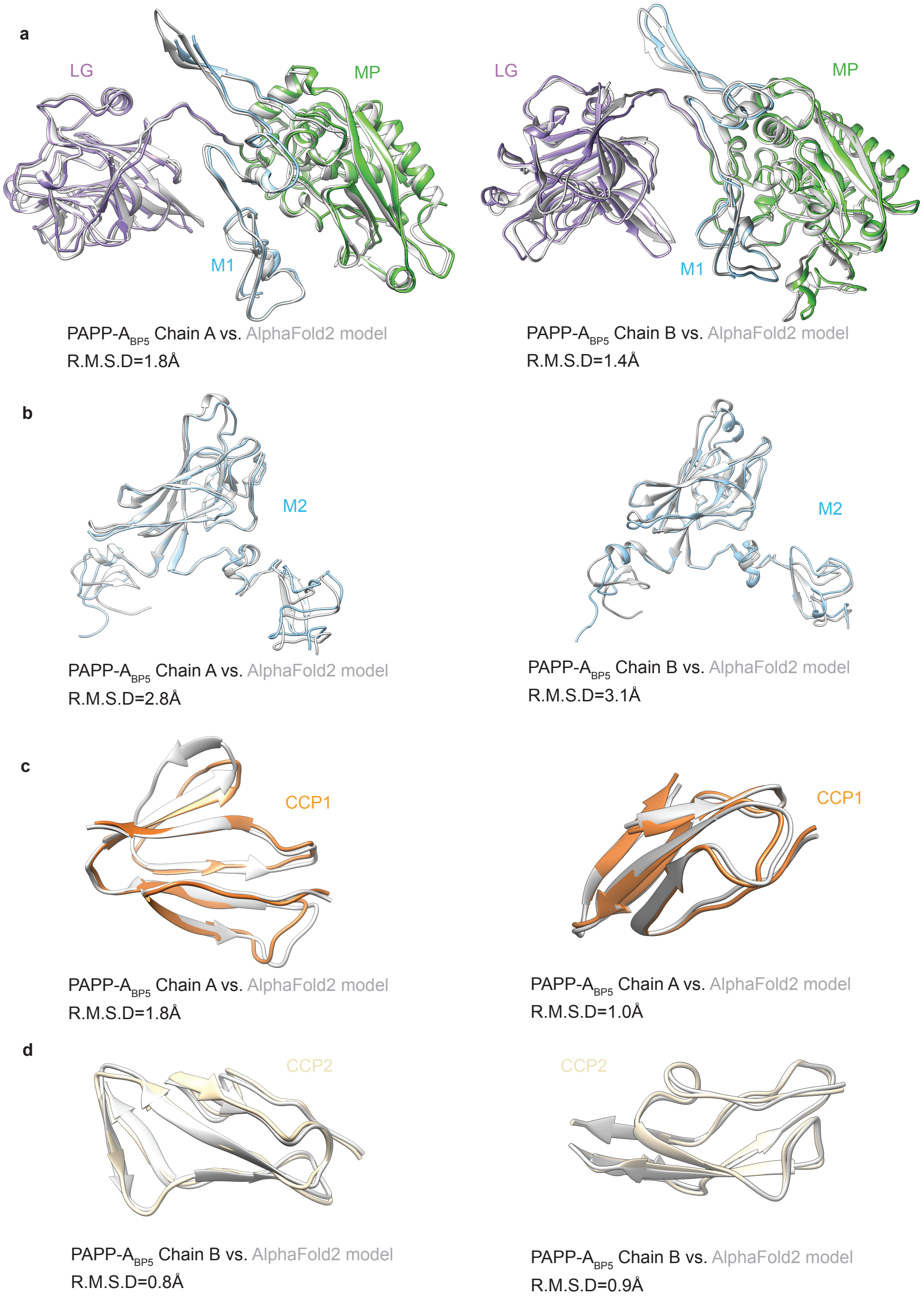

Supplementary Fig. 8: Experimental PAPP-A$_{BP5}$ structure alignment with AlphaFold predicted model
a, Overlay of PAPP-A$_{BP5}$ cryo-EM structure and AlphaFold prediction (light grey), with a focus on Laminin G-like (LG) domain (purple), metalloprotease (MP) domain (green) and central M1 domain (light blue). **b**, Overlay of PAPP-A$_{BP5}$ cryo-EM structure and AlphaFold prediction, with a focus on M2 domain (light blue). **c**, Overlay of PAPP-A$_{BP5}$ cryo-EM structure and AlphaFold prediction, with a focus on CCP1 domain (chocolate). **d**, Overlay of PAPP-A$_{BP5}$ cryo-EM structure and AlphaFold prediction, with a focus on CCP2 domain (wheat). In **a-d,** structure alignments of chain A were shown on the left panel and the alignments of chain B were shown on the right panel. The AlphaFold model is shown in cartoon in light grey, and different domains of PAPP-A in experimental PAPP-A$_{BP5}$ cryo-EM structure were colored by corresponding colors as **Fig. 1a**. The alignments suggest that AlphaFold prediction largely agrees with the experimental PAPP-A$_{BP5}$ cryo-EM structure, with low R.M.S.D value. M2 domain shows a higher R.M.S.D value compared with other domains, as the dimerization region within M2 has a larger difference.

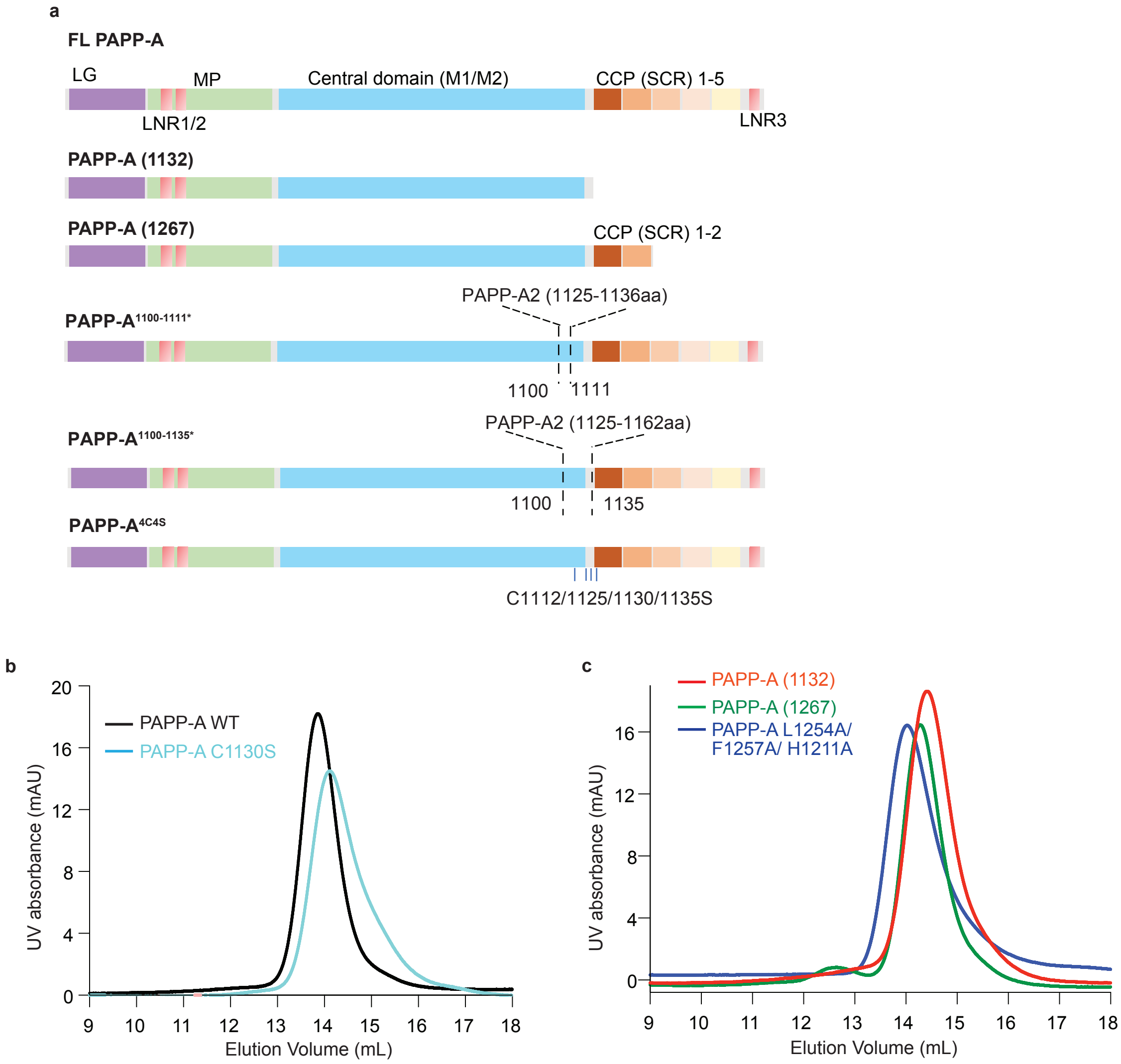

Supplementary Fig. 9: PAPP-A C-terminal truncation and chimera constructs used in the study
a, Schematic diagram of different PAPP-A constructs used in the study. Laminin G-like domain (LG) is colored in purple, metalloprotease domain (MP) in green, central domain (M1/M2) in light blue, the Complement Control Protein (CCP) domains including CCP1 in chocolate, CCP2 in wheat, CCP3 in peach puff, CCP4 in misty rose, CCP5 in khaki and the Lin12-Notch Repeats (LNR1/2 and LNR3) in salmon. **b**, PAPP-A C1130S mutant does not disrupt dimer formation, analyzed by size-exclusion chromatography. **c**, PAPP-A dimer formation is independent of LG-CCP2 domain interaction. Size-exclusion chromatography of purified recombinant protein of PAPP-A (1132), PAPP-A (1267) and PAPP-A L1254A/F1256A/H1211A showed that the three PAPP-A variants were both purified as auto-cleaved products and eluted out as dimers from the column. The SEC assays in **b-c** were performed at 1.4μM protein concentration. Source data are provided as a source data file.

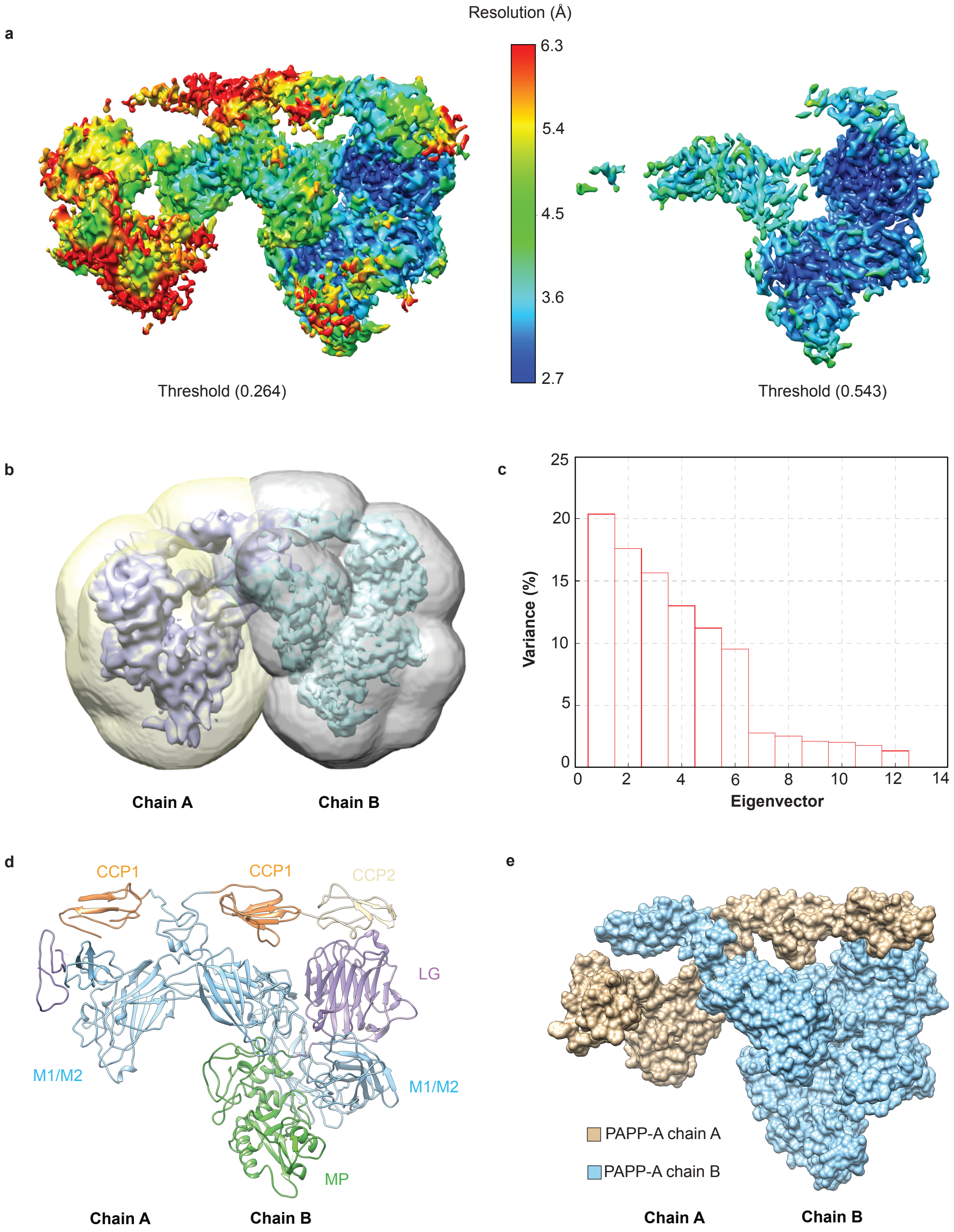

Supplementary Fig. 10: Cryo-EM structure determination of substrate-unbound PAPP-A
a, Cryo-EM density map of the substrate-unbound PAPP-A complex at an overall resolution of 3.35Å (EMD-27253, [https://www.ebi.ac.uk/emdb/EMD-27253]), with two threshold levels used to highlight local resolution (left: 0.264 and right: 0.543). The structure showed that one monomer exhibits a much higher resolution than the other and the PAPP-A core-domains have higher resolution than the C-terminal domains. The 3D cryo-EM map is colored by resolution (bar in the middle). The local resolution is calculated by cryoSPARCv3. **b**, Two soft-body masks covering each half of the density map were used for multi-body analysis. **c**, Eigenvector analysis for multi-body motion indicates the two PAPP-A chains twist relative to each other. Eigenvector analysis for multi-body motion indicates that 20.3% of variance is due to first 1 eigenvector (Supplementary Movie 2) with the first 6 accounting for 87.4% of variance. **d**, Substrate-unbound PAPP-A complex structure represented in ribbon (PDB 8D8O, [https://www.rcsb.org/structure/8D8O]) with PAPP-A domains highlighted in corresponding colors (Laminin G-like (LG) purple, metalloprotease (MP) green, the central M1/M2 light blue and the Complement Control Protein domains CCP1 chocolate, CCP2 wheat). **e**, Overall structure of substrate-unbound PAPP-A in surface representation showed that PAPP-A exists as a dimer in trans. PAPP-A chain A and chain B are colored in wheat and blue, respectively.

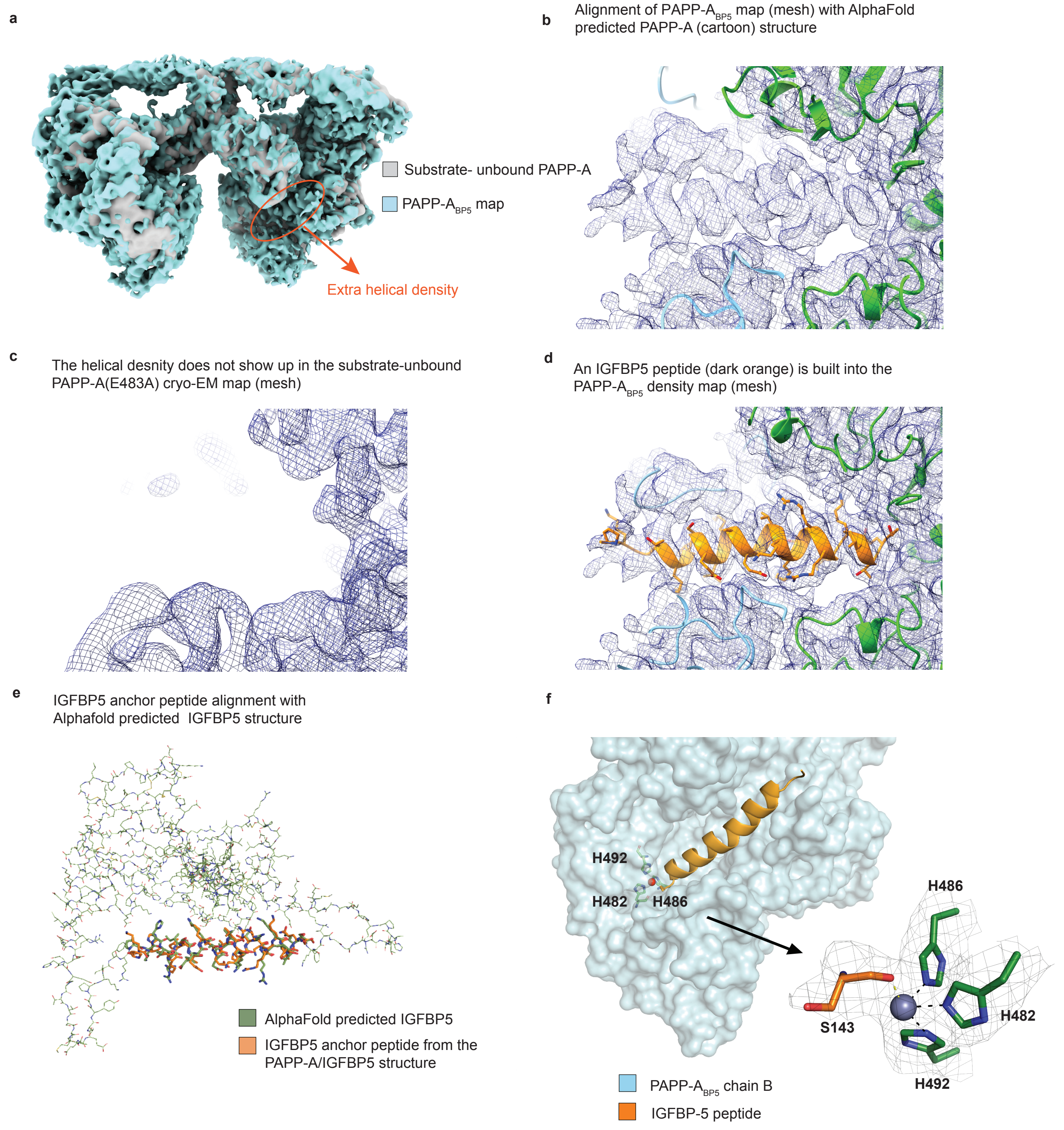

Supplementary Fig. 11: PAPP-A$_{BP5}$ complex structure revealed an IGFBP5 peptide helical density
a, Overlay of substrate-free PAPP-A cryo-EM density map (grey) and PAPP-A$_{BP5}$ complex cryo-EM map (light blue) showed that PAPP-A$_{BP5}$ has an extra helical density (highlighted in orange ellipse) that is not present in the substrate-unbound PAPP-A (E483A) map. **b**, Compared with AlphaFold predicted structure, the PAPP-A$_{BP5}$ complex cryo-EM map revealed an extra 6-turn α-helical density that sits between the metalloprotease (MP) domain (green) and central (M1/M2) domain (light blue). The cryo-EM map is shown as mesh density and predicted structure in cartoon representation. **c**, The substrate-unbound PAPP-A (E483A) cryo-EM density map does not contain this α-helical density. **d**, An IGFBP5 peptide (residues 119 to 143) can be built into the α-helical density. The IGFBP5 peptide is highlighted in dark orange. **e**, Overlay of AlphaFold predicted FL IGFBP5 (dark green) with IGFBP5$^{119\text{-}143}$ anchor peptide (dark orange) determined by cryo-EM. The predicted and experimentally determined IGFBP5$^{119\text{-}143}$ align well. The periphery regions show large flexibility in the predicted structure. **f**, Overview of the IGFBP5 anchor peptide binding in the PAPP-A grove formed by both the metalloprotease and central domains. IGFBP5 peptide shown as a ribbon in dark orange. The peptide binds closely to the active site, and the three histidine residues mediating the interaction with the zinc ion (ball density) are highlighted by sticks. (Right) Enlarged zinc ion interaction with three histidines and S143. Cryo-EM density is depicted by the gray mesh surface and fitted residues as sticks.

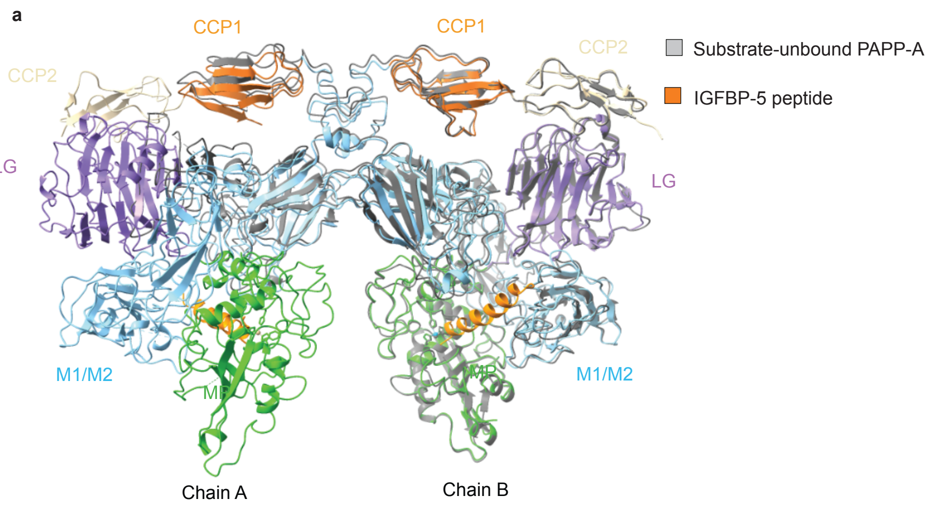

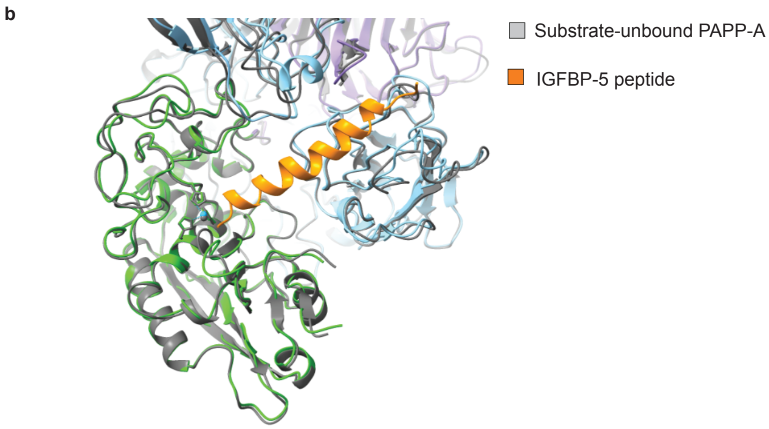

Supplementary Fig. 12: Structure comparison of substrate-unbound PAPP-A and PAPP-A$_{BP5}$ complex
a, Overlay of substrate-unbound PAPP-A cryo-EM structure (grey) and PAPP-A$_{BP5}$ complex. Different domain highlighted by corresponding colors: Laminin G-like domain (LG) purple, metalloprotease domain (MP) green, central domain (M1/M2) light blue, the Complement Control Protein domains (CCP1) chocolate and (CCP2) wheat. Although only partial structure was reconstituted for chain A in the substrate-unbound structure, the alignment of chain B of the two structures suggests no significant conformational changes were introduced upon association with the IGFBP5 anchor peptide (dark orange). **b**, Structure comparison of substrate-unbound and substrate-bound PAPP-A, with enlarged IGFBP5 anchor peptide binding pocket. The two structures largely agree with each other, suggesting the substrate association does not introduce substantial conformational changes on PAPP-A. There seems to be slight differences in a few loops, but this may due to the limited resolution and the inherent flexibility of these loops.

a

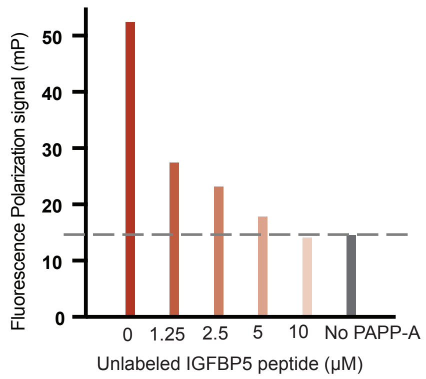

b

IGFBP5 residues	PAPP-A residues	Type of interaction
S125	N687	Hydrogen bond
I127	F722, Y1030, I976, K674	Hydrophobic
K128	H781, backbone carbonyl of N683	Hydrogen bond
K128	W658	Hydrophobic
A131	W658	Hydrophobic
V132	W658, H657, V677	Hydrophobic
K133	Backbone carbonyl of G1031	Hydrogen bond
T141	W451	Hydrophobic
Q142	Backbone nitrogen of A449	Hydrogen bond
S143	Carbonyl to catalytic Zn	Metal ion

c

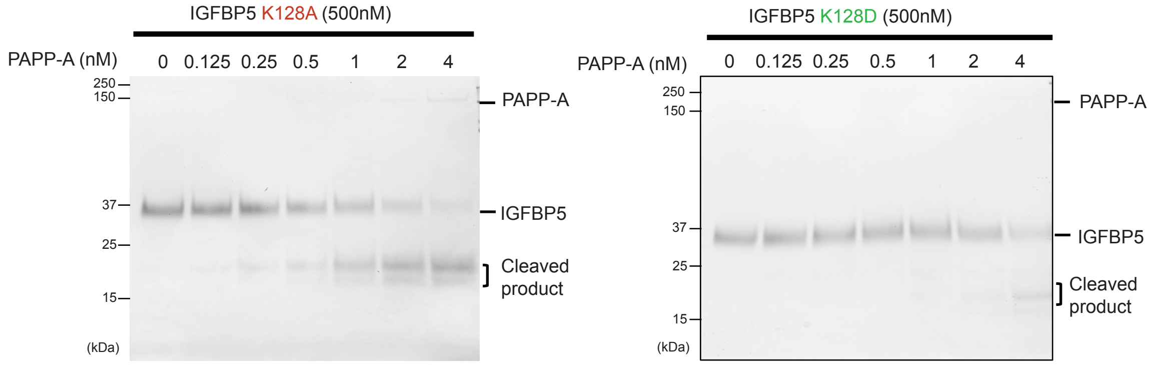

Supplementary Fig. 13: IGFBP5 interaction with PAPP-A

a, Fluorescence polarization (FP) competition assay with non-labeled IGFBP5 anchor peptide. 100nM WT PAPP-A, 5nM labeled peptide and the non-labeled $IGFBP5^{121\text{-}143}$ peptide in serial dilution were added to the reactions. Compared with labeled peptide alone control (dark grey, column on the right), the non-labeled peptide competed the binding with WT PAPP-A in a dose-dependent manner, and 10μM non-labeled peptide outcompetes the labeled peptide binding with WT PAPP-A. The FP values were recorded at 10 min. **b**, Table of interaction pairs between PAPP-A and IGFBP5 revealed by the $PAPP\text{-}A_{BP5}$ cryo-EM structure. **c**, Comparison of WT PAPP-A proteolytic activity towards WT IGFBP5, IGFBP5 K128A and IGFBP5 K128D as illustrated in the gel-based assay. The residue K128 was shown to be crucial for PAPP-A efficient recognition, as mutations either to Ala or Asp impair PAPP-A's recognition for IGFBP5. 500nM WT IGFBP5 or mutants were incubated with WT PAPP-A in dose-response. The reactions were performed at 37°C for 4 hours. Data shown was one representative of 3 independent replicates. Source data are provided as a source data file.

a

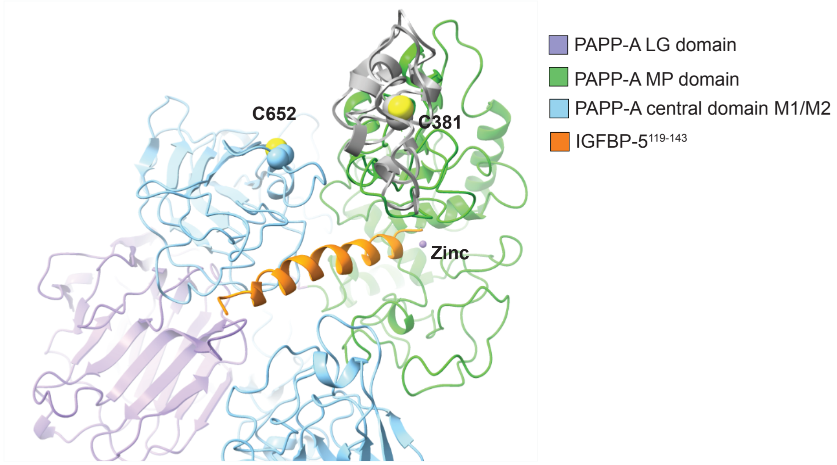

b

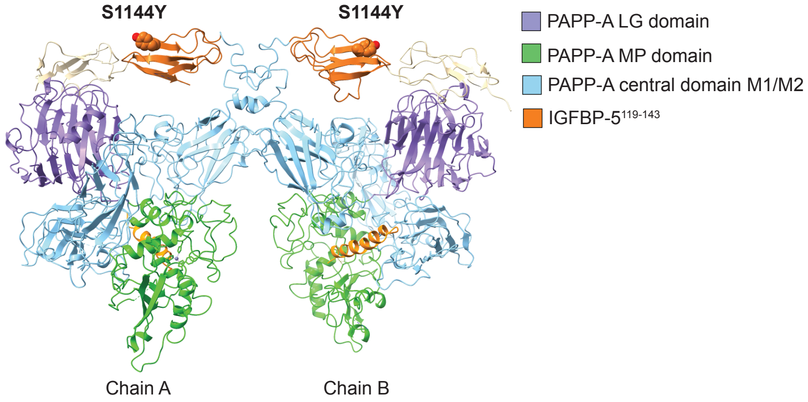

Supplementary Fig. 14: Some important PAPP-A residues identified from previous studies
a, Position of Cys652 and Cys381 that are reported to form disulfide bonds with proMBP (20). Cys652 resides on a loop that is proximate to the IGFBP5 binding groove. The AlphaFold model (grey) is used to illustrate the expected position of C381 as this region was not observed in the experimental cryo-EM structure. The cysteine residues are highlighted as spheres. PAPP-A_{BP5} structure is shown in cartoon representation (Laminin G-like domain (LG) purple, metalloprotease domain (MP) green, central domain (M1/M2) light blue), with the IGFBP5 anchor peptide highlighted in dark orange. **b**, Position of residue Y1144 (rs7020782 SNP in PAPP-A) (41) on the determined PAPP-A_{BP5} structure, with domains colored as in (**a**), with CCP1 in chocolate and CCP2 in wheat.

```
                    Signal sequences of IGFBPs

P24592|IGFBP6      -----------MTPHRLLPPLLLLLALLLA---------ASPGGALARCPGCGQGVQAGC
P18065|IGFBP2      MLPRVGCPALPLPPPPLLPLLLL-LL------GASGGGGGARAEVLFRCPPCTPERLAAC
P17936|IGFBP3      ---------MQRARPTLWAAALTLLVLLRGPPVARAGASSAGLGPVVRCEPCDARALAQC
P22692|IGFBP4      ----------------MLPLCLVAAL------LLAAGPGPSLGDEAIHCPPCSEEKLARC
P24593|IGFBP5      --------------MVLLTAVLLLA---------AYAGPAQSLGSFVHCEPCDEKALSMC
P08833|IGFBP1      --------MSEVPVARVWLVLLLLTV-----------QVGVTAGAPWQCAPCSAEKLALC
consensus                     .    .....*....         ... .  .. ...*..*. .... *

P24592|IGFBP6  14  PG------------------GCVEEEDGGSPAEGCAEAEGCLRREGQECGVYTPNCAPGL
P18065|IGFBP2  19  GPPPVAPPAAVAAVAGGARMPCAEL----VREPGCGCCSVCARLEGEACGVYTPRCGQGL
P17936|IGFBP3  25  APPP---------------AVCAEL----VREPGCGCCLTCALSEGQPCGIYTERCGSGL
P22692|IGFBP4  18  RPP----------------VGCEEL----VREPGCGCCATCALGLGMPCGVYTPRCGSGL
P24593|IGFBP5  19  PPSP---------------LGC-EL----VKEPGCGCCMTCALAEGQSCGVYTERCAQGL
P08833|IGFBP1  17  PPVS---------------ASCSEV----TRSAGCGCCPMCALPLGAACGVATARCARGL
consensus          ....               ..* *.    ....**... .*.. .*. **..*..*. **

P24592|IGFBP6  56  QCHPPKDDEAPLRALLLGRGRCLPARAPAVAEENPKESKPQAGTARPQDV----------
P18065|IGFBP2  75  RCYPHPGSELPLQALVMGEGTCEKRRDAEYGASPEQVADN---GDDHSEGGLVENHVDST
P17936|IGFBP3  66  RCQPSPDEARPLQALLDGRGLCVNASAVSRLRAYLLPAPPAPGNASESEEDRSAGSVESP
P22692|IGFBP4  58  RCYPPRGVEKPLHTLMHGQGVCMELAEIEAIQESLQPSDK-------DEGDHPNNSF---
P24593|IGFBP5  59  RCLPRQDEEKPLHALLHGRGVCLNEKSYREQVKIER-------DSREHEEPT-------T
P08833|IGFBP1  58  SCRALPGEQQPLHALTRGQGACVQESDASAPHAAEAGSPESPESTEITEEELLDNFH---
consensus          .* . . ...**..*..*.*.*.. ..       .  .         ....   .

P24592|IGFBP6 106  ---------------------------------NRRDQQRN----PGTSTTPSQPNSAGV
P18065|IGFBP2 132  MNMLG-GGGSAGRKPLKSGMKELAV------FREKVTEQHRQMGKGGKHHLGLEEPKKLR
P17936|IGFBP3 126  SVSSTHRVSDPKFHPLHSKIIIIKKGHAKDSQRYKVDY-------ESQSTDTQNFSSESK
P22692|IGFBP4 108  ----S-PCSAHDRRCLQ---KHFAK------IRD-------RSTSGGKMKVNG-APREDA
P24593|IGFBP5 105  SEMAEETYSPKIFRPKHTRISELKAEAVKKDRRKKLTQSKFVGGAENTAHPRIISAPEMR
P08833|IGFBP1 110  -LMAP---SEEDHSILWDAIS----------------------TYDGSKAL-----HVTN
consensus            .   .     ...... .  .         . ..   .     ... ..     .. .

P24592|IGFBP6 129  QDTEMGPCRRHLDSVLQQLQTEVYRG-------AQTLYVPNCDHRGFYRKRQCRSSQGQR
P18065|IGFBP2 185  PPPARTPCQQELDQVLERISTMRLPDERGPLEHLYSLHIPNCDKHGLYNLKQCKMSLNGQ
P17936|IGFBP3 179  RETEYGPCRREMEDTLNHLKFLNVLS-------PRGVHIPNCDKKGFYKKKQCRPSKGRK
P22692|IGFBP4 146  RPVPQGSCQSELHRALERLAAS----QSRTHEDLYIIPIPNCDRNGNFHPKQCHPALDGQ
P24593|IGFBP5 165  QESEQGPCRRHMEASLQELKASPRMV-------PRAVYLPNCDRKGFYKRKQCKPSRGRK
P08833|IGFBP1 144  IKKWKEPCRIELYRVVESLAKA----QETSGEEISKFYLPNCNKNGFYHSRQCETSMDGE
consensus           ... ..*..... .....            . .  ...***...*.....**.......

P24592|IGFBP6 182  RGPCWCVDR-MGKSLPGSPDGN-GSSSCPTGSSG-------------
P18065|IGFBP2 245  RGECWCVNPNTGKLIQGAPTIR-GDPECHLFYNEQQEARGVHTQRMQ
P17936|IGFBP3 232  RGFCWCVDK-YGQPLPGYTTKGKEDVHCYSMQSK-------------
P22692|IGFBP4 203  RGKCWCVDRKTGVKLPGGLEPK-GELDCHQLADSFRE----------
P24593|IGFBP5 218  RGICWCVDK-YGMKLPGMEYVD-GDFQCHTFDSSNVE----------
P08833|IGFBP1 200  AGLCWCVYPWNGKRIPGSPEIR-GDPNCQIYFNVQN-----------
consensus          .* ****..  *....* .... ..  *... . . .
```

Supplementary Fig. 15: Sequence alignment of IGFBP 1-6

Multiple sequence alignment of human IGFBP proteins using Clustal Omega (version 1.2.4; Supplementary reference 3). The signal sequences of IGFBPs were highlighted by a yellow background. Black and gray boxes highlight identical and similar residues, respectively. The IGFBP5 anchor peptide is highlighted in orange, and only IGFBP3 shows sequence conserva-tion for this anchor region. Highlighted in yellow within the orange sequence is the anchoring lysine residue K128 in IGFBP5. The specific PAPP-A cleavage site residues of IGFBP4 and IGFBP5 were highlighted in red, and the cleavage site is labeled by green arrow. All IGFBPs are numbered after signal sequences.

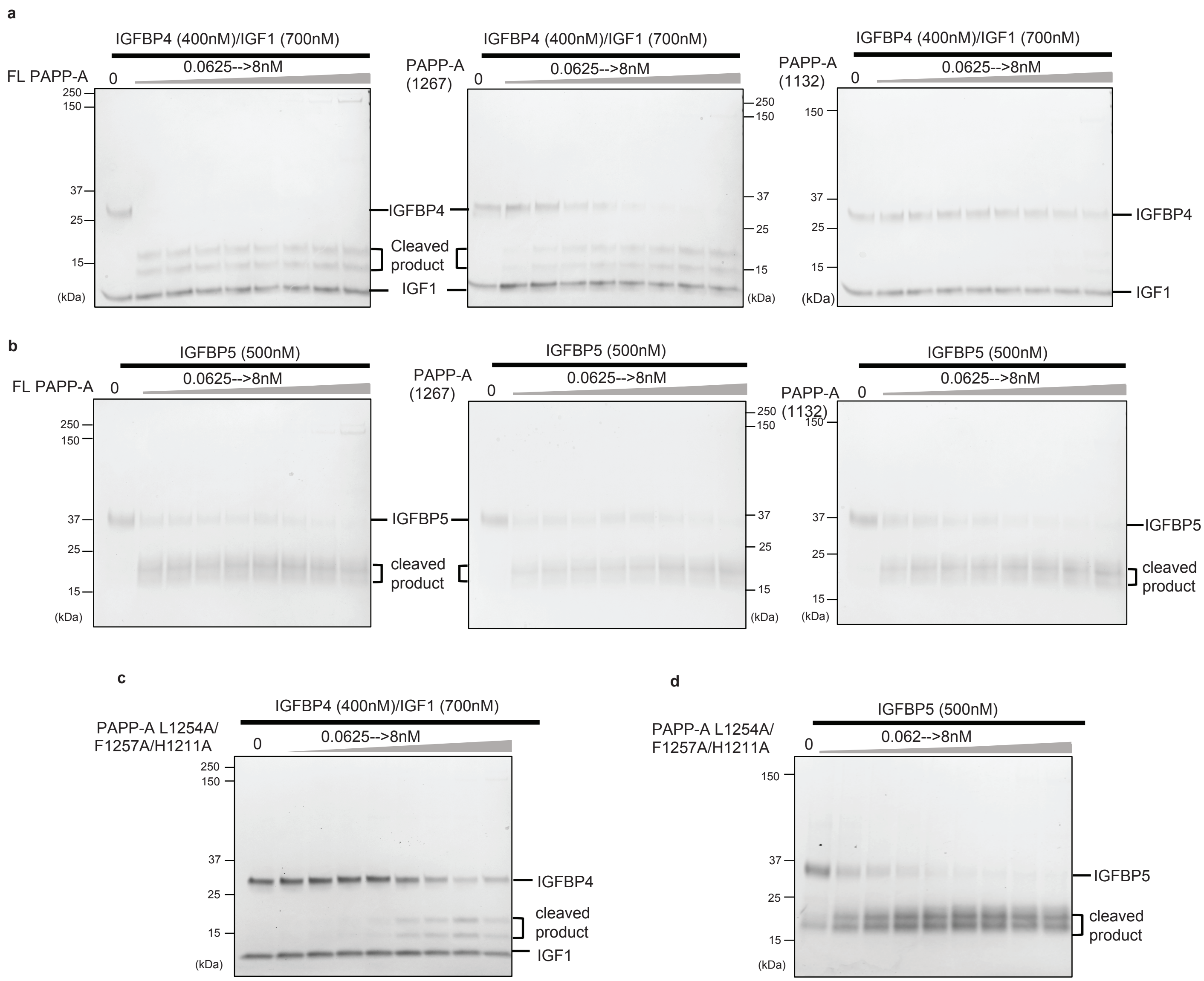

Supplementary Fig. 16: Interaction between Laminin G (LG)-like domain and CCP2 is important for PAPP-A's proteolytic activity towards IGFBP4, but not IGFBP5

a-b, Comparison of IGFBP4 and IGFBP5 cleavage between WT FL PAPP-A and PAPP-A C-terminal truncations in the gel-based assay. **a**, IGFBP4 was fully cleaved by WT FL PAPP-A under pM concentration in an IGF1-dependent manner, whereas IGFBP4 was not responsive with PAPP-A (1132). PAPP-A (1267) significantly rescued the proteolytic activity for IGFBP4. **b,** In comparison, all three PAPP-A constructs have similar proteolytic activity towards IGFBP5, suggesting IGFBP5 cleavage by PAPP-A is independent of the CCP2 and LG domain interaction. **c-d**, Proteolytic activity assay with PAPP-A mutation L1254A/F1257A/H1211 towards IGFBP4 and IGFBP5. Compared with WT PAPP-A in (**a**), the triple mutation significantly reduced the proteolytic activity for IGFBP4 (**c**), while it retains similar IGFBP5 proteolytic activity as WT PAPP-A (**d**). In (**a**) and (**c**), IGF1 was included for the IGFBP4 proteolytic activity assay. The pre-incubated IGFBP4/IGF1, or IGFBP5 were mixed with WT PAPP-A, PAPP-A C-terminal truncations, or triple mutant in a 2-fold serial-dilution (8nM to 62.5pM), at 37°C for 4 hours. Concentrations of WT PAPP-A and PAPP-A variants used in the assay were calculated by the dimer molecular weight. Data shown in **a-d** were representatives of 3 independent replicates. Quantitative comparison was performed across samples from the same experiment with gels run in parallel. Source data are provided as a source data file.

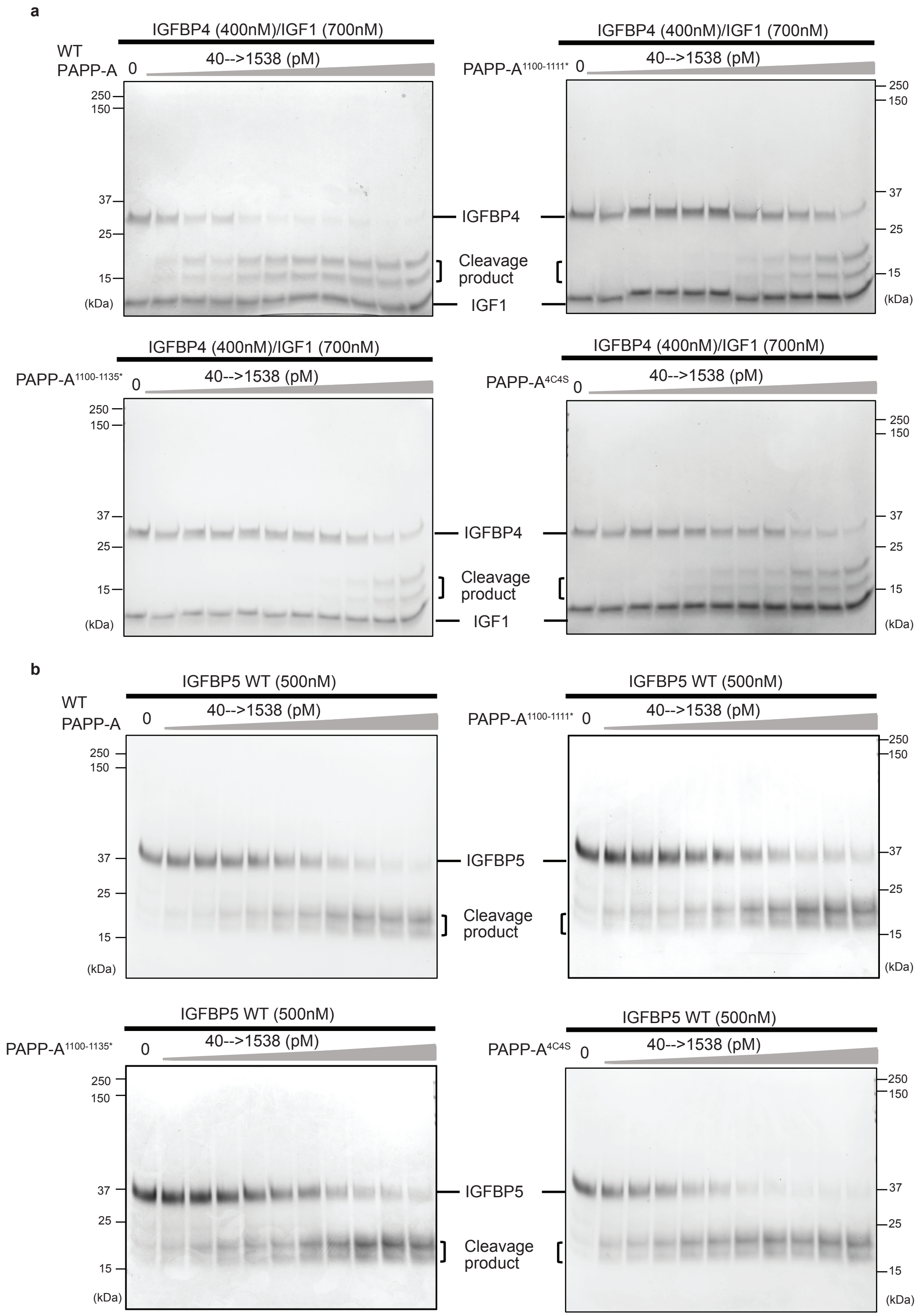

Supplementary Fig. 17: PAPP-A dimer formation is necessary for IGFBP4 cleavage, but not for IGFBP5
a-b, Gel-based proteolytic activity of WT PAPP-A and PAPP-A monomeric variants towards IGFBP4 and IGFBP5. Pre-incubated IGFBP4/IGF1 or IGFBP5 were mixed with WT PAPP-A or PAPP-A monomeric variants in a serial dilution (1538pM to 40pM) at 37°C for 4 hours. In a, all three PAPP-A monomer variants appear to have decreased IGFBP4 cleavage activity, compared with WT PAPP-A. In **b**, the IGFBP5 cleavage seems to be independent of PAPP-A dimer formation. Concentrations of WT PAPP-A and PAPP-A monomer variants used in the assay were calculated by the monomer molecular weight. Data shown in **a-b** were representatives of 3 independent replicates. Quantitative comparison was performed across samples from the same experiment with gels run in parallel. Source data are provided as a source data file.

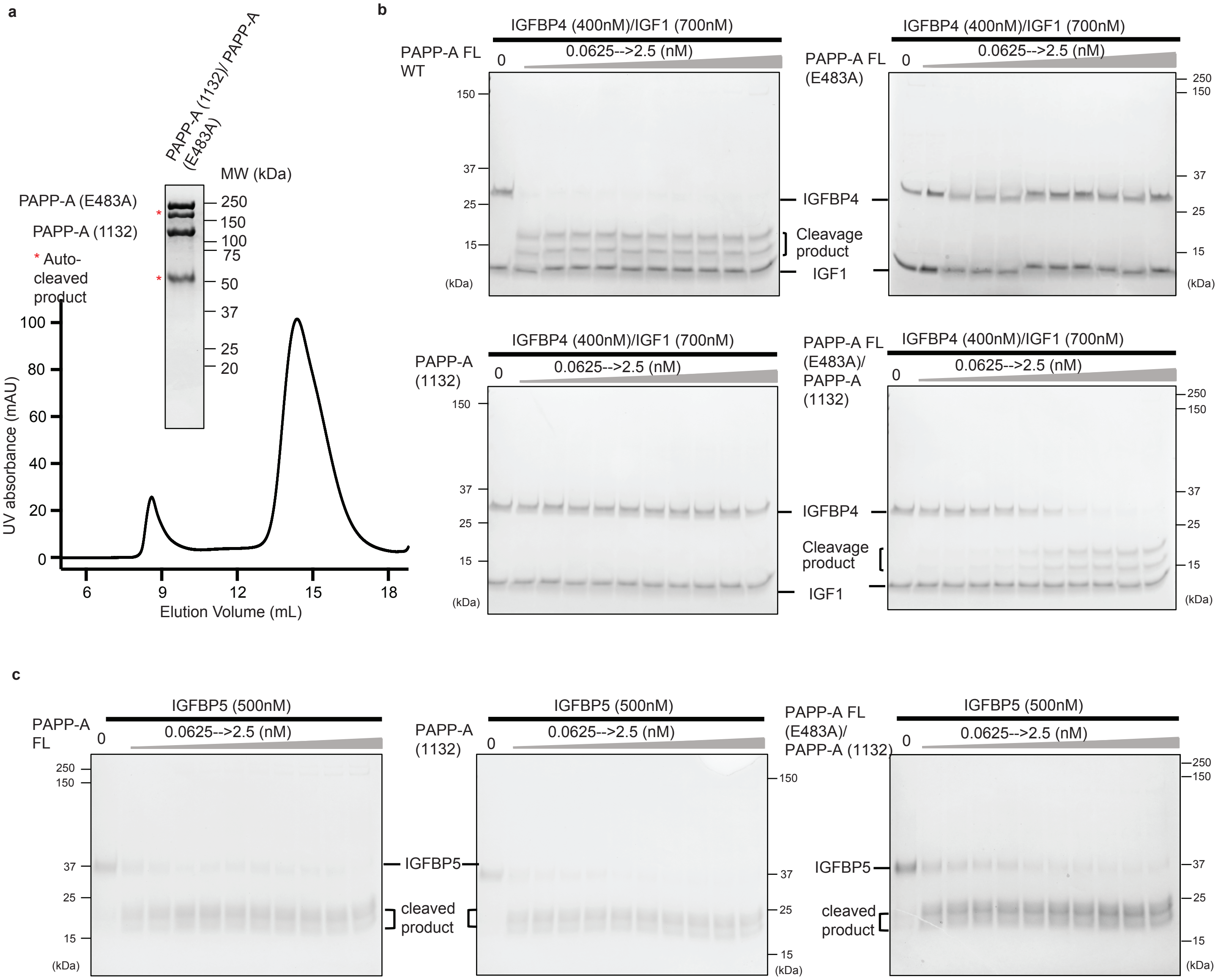

Supplementary Fig. 18: PAPP-A exists as a dimer in *trans*, validated by the co-expressed PAPP-A heterodimer
a, Size-exclusion chromatography of the co-expressed PAPP-A (E483A)/PAPP-A (1132). The two chains co-eluted from the column, with the elution volume agreeing with a heterodimer position. UV absorbance chromatograms as well as Coomassie-stained fractions are shown. **b-c**, Proteolytic activity assay with co-expressed PAPP-A (E483A)/-PAPP-A (1132) for IGFBP4 and IGFBP5. In **b**, the co-expressed PAPP-A (E483A)/PAPP-A (1132) showed significant gain-of-function towards IGFBP4 cleavage, compared with PAPP-A (E483A) or PAPP-A (1132), although the activity is still lower than that of WT PAPP-A. In **c**, the co-expressed PAPP-A (E483A)/PAPP-A (1132) heterodimer has similar cleavage activity for IGFBP5 as the WT FL PAPP-A homodimer and PAPP-A (1132). The reactions in **b-c** were performed in a protease serial-dilution (2.5nM to 62.5pM) at 37°C for 4 hours. Concentrations of WT PAPP-A, PAPP-A (E483A), PAPP-A monomer variants and PAPP-A heterodimer used in the assay were calculated by the dimer molecular weight. Data shown in **b-c** are representative of 3 independent replicates. Quantitative comparison was performed across samples from the same experiment with gels run in parallel. Source data are provided as a source data file.

a

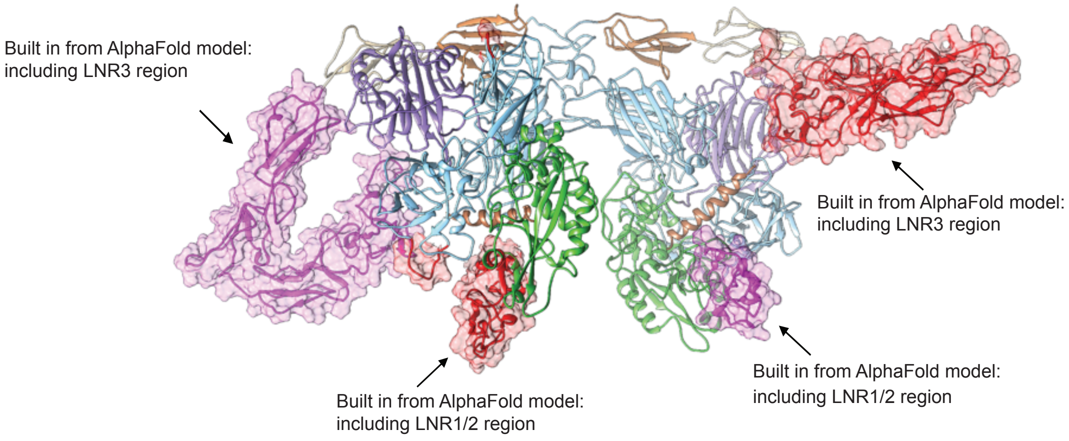

b

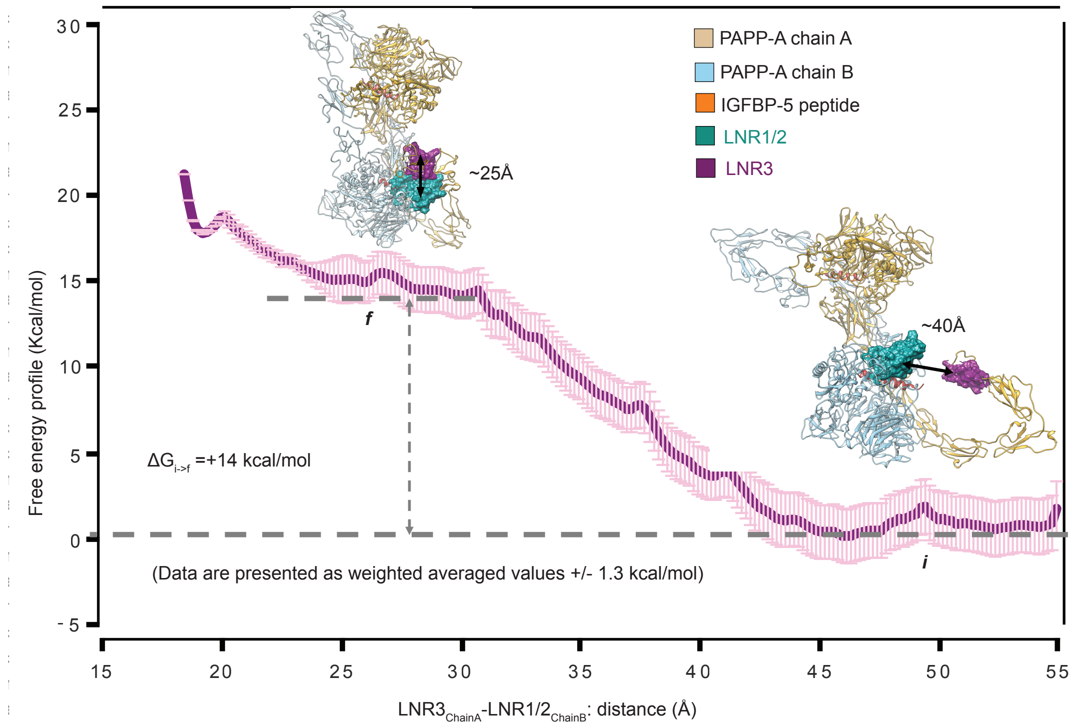

Supplementary Fig. 19: Analysis of Lin12-Notch Repeat interaction (LNR1/2 with LNR3) by molecular dynamic simulations

a, Our complete model of PAPP-A homodimer by building in the missing regions to the cryo-EM protein construct using the PAPP-A monomer predicted from AlphaFold. PAPP-A determined by Cryo-EM is colored by domains: Laminin G-like domain in purple, metalloprotease domain in green, central domain M1/M2 in light blue, the Complement Control Protein domains CCP1 in chocolate and CCP2 in wheat. For regions built in from the AlphaFold model, the domains are shown in both cartoon and surface representation, highlighted by red and magenta. **b,** Affinity free energy between LNR1/2 of chain B (the center of mass of Cas for residues 335-394) and LNR3 of chain A (the center of mass of Cas for residues 1478-1504) using umbrella sampling method. We considered 17 separate windows/conformations with 2 Å increment in distance between $LNR1/2_{ChainB}$ and $LNR3_{ChainA}$, varying from 19 Å to 53 Å. We performed a set of 5.2 ns MD simulation on each window for the free energy estimation. The weighted histogram analysis method (Supplementary reference 17) was used to obtain the weighted averaged values of free energy. The statistical errors (S.E) were evaluated by the bootstrap method (Supplementary reference 18) using 100 rounds of bootstrapping analysis as shaded with pink.

Supplementary Table 1: Cryo-EM data collection, refinement and validation statistics

	PAPP-A$_{BP5}$ (EMDB-26475) (PDB 7UFG)	PAPP-A (EMDB-27253) (PDB 8D8O)
**Data collection and processing**		
Magnification	105,000	105,000
Voltage (kV)	300	300
Electron exposure (e–/Å^2)	46.8-48.3	46.8-48.4
Defocus range (μm)	-1.0 – 3.5	-1.0 – 3.5
Pixel size (Å)	0.83	0.83
Symmetry imposed	C1	C1
Initial particle images (no.)	9,080,999	4,032,492
Final particle images (no.)	241,885	338,320
Map resolution (Å)	3.28	3.35
FSC threshold	0.143	0.143
Map resolution range (Å)	2.7 – 7.9	2.7 – 7.9
**Refinement**		
Initial model used (PDB code)	AlphaFold AF-Q13219	AlphaFold AF-Q13219
Map sharpening *B* factor (Å^2)	-100	-132.6
Model composition		
Non-hydrogen atoms	18,245	11,819
Protein residues	2,378	1,536
Ligands	2	1
B factors (Å^2)		
Protein	85.93	69.26
Ligand	105.91	56.61
R.m.s. deviations		
Bond lengths (Å)	0.003	0.004
Bond angles (°)	0.908	0.572
Validation		
MolProbity score	1.98	1.82
Clashscore	11.60	7.63
Poor rotamers (%)	0.05	0.23
Ramachandran plot		
Favored (%)	93.94	94.10
Allowed (%)	6.06	5.90
Disallowed (%)	0.00	0.00

Supplementary Table 2: Primer sequences used for cloning

	Constructs	Primers
**IGFBP5**	WT IGFBP5	F: 5’-GTCAGCGGCCGCCACCATGCTGCCTCTCTGCCTCGTGGCT-3’
		R: 5’-GCATGGCGCGCCTCACTTGTCGTCGTCGTCCTTGTAGTCAGCAGCGG-3’
	K128A	F: 5’-ACACACCCGCATCTCCGAGCTGGCCGCTGAAGCAGTGAAGAAGGACCG-3’
		R”5’-TGGGCCGGAAGATCTTGGGGGAGTAGGTCTCCTCGGCCATCTCAGAGGT-3’
	K128D	F: 5’-CACACCCGCATCTCCGAGCTGGACGCTGAAGCAGTGAAGAAGGACCG-3'
		R: 5’-TTTGGGCCGGAAGATCTTGGGGGAGTAGGTCTCCTCGGCCATCTCAGAG-3’
	IGFBP5$^{121-143}$ replaced by IGFBP4$^{114-134}$	F: 5’-TGCGAAGATCAGGGATAGAAAGTTTGTCGGGGGAGCCGAGAACACTGCCC-3’
		R: 5’-AAATGCTTTTGCAAGCATCTTTTGGGCCGGAAGATCTTGGGGGAGTAGGT-3’
**PAPP-A**	WT PAPP-A	3 Synthetic genes IDT gBlocks
	PAPP-A E483A	F: 5’-GCCATACACACACCATGATCCACGCCATAGGCCATTCTCTGGGACTCTA-3’
		R: 5’-CGGGCATCCCATAAAAAGAGGGATTGAGACCGCGGCCGCCCAGCTTCTA-3’
	PAPP-A$^{1100-1111*}$	F1: 5’-GCATCGTCTCTGCGCAAAGCGACCCCGGAGATTGCAACGAGGGGAC-3’
		F2: 5’-GCGCACTTCATCCCGGATTGGACTGAGCGCACCATCAAAC-3’
		R1: 5’-GCTACGTCTCATCATGTCAACGGGGCGAAACCTATA-3’
		R2: 5’-ATGAGTTTGATGGTGCGCTCAGTCCAATCCGGGATGAAGT-3’
	PAPP-A$^{1100-1135*}$	F1: 5’-FGCATCGTCTCTGCGCAAAGCGACCCCGGAGATTGCAACGAGGGGAC-3’
		F2: 5’-GCTACGTCTCAGCGCACATCCTCCCGCATTGGTCTTTCGG-3’
		R1: 5’-GCTACGTCTCACCAGAGCTCGCTGTAGAAAATGCTTACTTGAACTG-3’
		R2: 5’-GCATCGTCTCTCTGGACAGCTGTCCTGCTTCCCACAGGGC-3’
	PAPP-A^{4C4S}	F:5’-CCCCGCAGAGCAGTCATCTGTGCACTTCGCATCTGAGAAAACAGATTCCCCAGAGCTCGC-3’
		R: 5’-CTATAGGTTTCGCCCCGTTGAGATGATGACAAGGTGACGGGGTCGAAGTTGTCAAAGGAG-3’
	PAPP-A (1132)	F: 5’-GCCTTTCTCTCCACAGGTGTCCACTCCCAGGTCCAAGTTT-3’
		R: 5’-GCATGGTCTCTCCCTTTCTCACATGCGAAGTGCACACATGACTGCT-3’
	PAPP-A (1267)	F: 5’-GCCTTTCTCTCCACAGGTGTCCACTCCCAGGTCCAAGTTT-3’
		R: 5’-GCATGGCGCGCCTTTACTTATCGTCGTCATCCTTGTAATCAAGGCACATCAGTTCACAGA-3’
	PAPP-A C1130S	F: 5’-AGCAGTCATGTGTGCACTTCGCAAGCGAGAAAACAGATTGCCCAGAGCT-3’
		R: 5’-CTGCGGGGCTATAGGTTTCGCCCCGTTGACATGATGACAAGGTGACGGG-3’
	L1254A/F1257A/H1211A	F1: 5’-GTCTGCTTACATGCATGGAGGACGGCCTCGCCTCTGCCCCTGAGGCCCTCTGTGAACTGA-3’
		F2: 5’-TGGACTGCTCTATCCCGGATCATGCCCAGGTCTACGCGGCGTCATTTTC-3’
		R1: 5’-TGTTGTTACCCTTAAGTTGCGCAGGGTGGCGACACTGGAATGAACACTGA-3’
		R2: 5’-CCGGTTCGCATGCAACCTGTTTGTTCCATTTGCCCTCTGTACAGGTAACT-3’

Supplementary references

1, Goddard, T. D. *et al.* UCSF ChimeraX: Meeting modern challenges in visualization and analysis. *Protein Sci.* **27**, 14–25 (2018)
2, Pettersen, E. F. *et al.* UCSF ChimeraX: Structure visualization for researchers, educators, and developers. *Protein Sci.* **30**, 70–82 (2021)
3, Sievers, F. *et al.* Fast, scalable generation of high-quality protein multiple sequence alignments using Clustal Omega. *Mol. Syst. Biol.* **7**, 539 (2011)
4, Punjani, A., Rubinstein, J. L., Fleet, D. J. & Brubaker, M. A. cryoSPARC: algorithms for rapid unsupervised cryo-EM structure determination. *Nat. Methods* **14**, 290–296 (2017)
5, Tunyasuvunakool, K. *et al.* Highly accurate protein structure prediction for the human proteome. *Nature* 596, 590–596 (2021)
6, Varadi, M. et al. AlphaFold Protein Structure Database: massively expanding the structural coverage of protein-sequence space with high-accuracy models. *Nucleic Acids Res*. 50, D439–D444 (2022)
7, Mastronarde, D. N. Automated electron microscope tomography using robust prediction of specimen movements. *J. Struct. Biol.* **152**, 36–51 (2005)
8, Asarnow, D., Palovcak, E., Cheng, Y. UCSF pyem v0.5.
9, Liebschner, D. *et al.* Macromolecular structure determination using X-rays, neutrons and electrons: recent developments in Phenix. *Acta Crystallogr D Struct Biol* **75**, 861–877 (2019)
10, Emsley, P., Lohkamp, B., Scott, W. G. & Cowtan, K. Features and development of Coot. *Acta Crystallogr. D Biol. Crystallogr.* **66**, 486–501 (2010)
11, Rossi, A. M. & Taylor, C. W. Analysis of protein-ligand interactions by fluorescence polarization. *Nat. Protoc.* **6**, 365–387 (2011)
12, Needleman, S. B. & Wunsch, C. D. A general method applicable to the search for similarities in the amino acid sequence of two proteins. *J. Mol. Biol.* **48**, 443–453 (1970)
13, Shapovalov, M. V. & Dunbrack, R. L., Jr. A smoothed backbone-dependent rotamer library for proteins derived from adaptive kernel density estimates and regressions. *Structure* **19**, 844–858 (2011)
14, Abraham. High performance molecular simulations through multi-level parallelism from laptops to supercomputers. *Software X*
15, Torrie, G. M. & Valleau, J. P. Nonphysical sampling distributions in Monte Carlo free-energy estimation: Umbrella sampling. *J. Comput. Phys.* **23**, 187–199 (1977)
16, Tribello, G. A., Bonomi, M., Branduardi, D., Camilloni, C. & Bussi, G. PLUMED 2: New feathers for an old bird. *Comput. Phys. Commun.* **185**, 604–613 (2014)
17, Kumar, S., Rosenberg, J. M., Bouzida, D., Swendsen, R. H. & Kollman, P. A. THE weighted histogram analysis method for free-energy calculations on biomolecules. I. The method. *Journal of Computational Chemistry* vol. 13 1011–1021 (1992)

18, Hub, J. S., de Groot, B. L. & van der Spoel, D. G_wham—A free weighted histogram analysis implementation including robust error and autocorrelation estimates. *J. Chem. Theory Comput.* **6**, 3713–3720 (2010)
19, Yu, Y. *et al.* Semi-automated Optimization of the CHARMM36 Lipid Force Field to Include Explicit Treatment of Long-Range Dispersion. *J. Chem. Theory Comput.* **17**, 1562–1580 (2021)
20, Bussi, G., Donadio, D. & Parrinello, M. Canonical sampling through velocity rescaling. *J. Chem. Phys.* **126**, 014101 (2007)
21, Parrinello, M. & Rahman, A. Polymorphic transitions in single crystals: A new molecular dynamics method. *J. Appl. Phys.* **52**, 7182–7190 (1981)
22, Essmann, U. *et al.* A smooth particle mesh Ewald method. *J. Chem. Phys.* **103**, 8577–8593 (1995)
23, Miyamoto, S. & Kollman, P. A. Settle: An analytical version of the SHAKE and RATTLE algorithm for rigid water models. *J. Comput. Chem.* **13**, 952–962 (1992)
24, Hess, B. P-LINCS: A Parallel Linear Constraint Solver for Molecular Simulation. *J. Chem. Theory Comput.* **4**, 116–122 (2008)
25, Abraham, M. J. *et al.* GROMACS: High performance molecular simulations through multi-level parallelism from laptops to supercomputers. *SoftwareX* **1-2**, 19–25 (2015)
